# Alveolar echinococcosis drives functional reprogramming of hepatic CD8^+^ T cells

**DOI:** 10.3389/fcimb.2026.1747682

**Published:** 2026-02-19

**Authors:** Jing Tang, Xiaoli Qin, Siyu Hou, Yan Huo, Peijiao Wu, Bingshuo Qian, Yazhou Zhu, Zihua Li, Yinqi Zhao, Yangyang Zhang, Tao Li, Wei Zhao

**Affiliations:** 1 School of Basic Medical Sciences, Ningxia Medical University, Yinchuan, China; 2 Department of Pediatrics, General Hospital of Ningxia Medical University, Yinchuan, China; 3 Ningxia Key Laboratory for Prevention of Common Infectious Diseases, Yinchuan, China; 4 Department of Hepatobiliary Surgery, General Hospital of Ningxia Medical University, Yinchuan, China

**Keywords:** alveolar echinococcosis (AE), CD8^+^ T-cell functional reprogramming, cytotoxic/effector-memory/exhausted T cells, dendritic cell–T cell interaction, hepatic immune microenvironment, single-cell RNA sequencing (scRNA-seq)

## Abstract

**Background:**

Alveolar echinococcosis (AE), caused by the larval stage of *Echinococcus multilocularis*, exhibits infiltrative, tumor-like behavior in the liver and persists within its tolerogenic immune environment. Although T cells are central to host defense, the stage-specific remodeling of their lineage states during AE remains unclear.

**Methods:**

A secondary AE infection model was established by portal vein injection of approximately 1,000 viable protoscoleces in C57BL/6 mice. Liver tissues collected at 3 days (3 dpi) and 3 months (3 mpi) post-infection were analyzed using single-cell RNA sequencing (scRNA-seq), flow cytometry, and multiplex immunofluorescence to characterize T-cell subset composition, transcriptional programs, and potential interactions with dendritic cells (DCs).

**Results:**

scRNA-seq of 78,290 high-quality cells identified 13 immune and non-immune populations and revealed strong temporal shifts in hepatic immunity. Early infection featured macrophage-driven inflammation with reduced T-cell proportions, whereas late infection showed marked expansion of both T cells and DCs. CD8^+^ T-cell profiling demonstrated the establishment of a diversified compartment composed of cytotoxic, effector-memory, and exhausted subsets. These subsets exhibited coordinated transcriptional remodeling, including upregulation of regulatory genes (*Btg1, Tnfaip3, Junb, Nr4a1*) and downregulation of early-induced inflammatory and metabolic genes, indicating adaptation to sustained antigen exposure. Spatial imaging further revealed ring-like accumulation of CD11c^+^ DCs around lesions with adjacent clustering of CD8^+^ T cells, and ligand–receptor analysis highlighted Thy1–Adgre5 as a prominent DC–T-cell interaction axis.

**Conclusions:**

AE infection drives a transition from acute inflammation to chronic immune regulation through extensive lineage diversification and functional reprogramming of CD8^+^ T cells. Spatially organized DC–T-cell interactions likely contribute to maintaining a regulated yet immunologically active microenvironment, providing insights for targeting chronic-stage immune responses in AE.

## 1 Introduction

Alveolar echinococcosis (AE), caused by infection with the larval stage of *Echinococcus multilocularis (E. multilocularis)*, is a life-threatening zoonotic disease with a predilection for the liver (Casulli et al., 2019). Humans become infected through ingestion of food or water contaminated with parasite eggs, after which the released oncospheres migrate via the portal circulation to the liver and initiate lesion formation. The metacestode proliferates by exogenous budding to form multilocular vesicles that progressively infiltrate surrounding hepatic tissue after it is established (Taratuto and Venturiello, 1997; Lundstrom-Stadelmann et al., 2025). These lesions display biological features resembling malignant tumors, leading AE to be described as a “parasitic cancer” (Woolsey and Miller, 2021; Jensenius et al., 2024). Due to the often silent early clinical course, AE is frequently diagnosed at advanced stages when extensive hepatic infiltration or extrahepatic spread has already occurred. The disease carries a 10-year mortality rate approaching 90% in the absence of treatment (Brunetti et al., 2010; Kamiyama, 2020). Although benzimidazole therapy suppresses parasite growth, it rarely achieves complete clearance and is also associated with hepatotoxicity with long-term use (Falagas and Bliziotis, 2007; Chai et al., 2021). Consequently, AE is considered one of the most challenging helminthic diseases worldwide and is listed by the World Health Organization as one of 17 neglected tropical diseases targeted for control or elimination by 2050 (Wen et al., 2019).

The liver provides a unique immunological milieu characterized by basal immune tolerance, which normally safeguards against chronic inflammation but also offers a permissive environment for persistent infection (Casulli et al., 2019). During AE, this tolerogenic setting becomes further reshaped, enabling the parasite to evade immune surveillance (Zheng and Tian, 2019). Previous studies have reported a stage-dependent shift from early Th1-dominated inflammatory responses toward Th2- and regulatory-skewed profiles during chronic infection (Wang et al., 2018; Gottstein et al., 2017). CD8^+^ T cells, which serve as key effector cells against intracellular pathogens, frequently acquire features of dysfunction or exhaustion under chronic antigen exposure (Hou et al., 2020). However, how the lineage states and functional programs of CD8^+^ T cells evolve across distinct stages of AE—and how these changes contribute to the establishment of chronic immune equilibrium—remains incompletely defined.

Dendritic cells (DCs), as the principal antigen-presenting cells in the liver, are also likely to shape T-cell fate during AE (Wang and Gottstein, 2016). Changes in DCs phenotype and signaling can direct T-cell polarization toward effector, memory, or regulatory pathways, yet their spatial organization and interaction with T-cell subsets within AE lesions have not been systematically explored. Likewise, a comprehensive single-cell–level assessment of DC–T cell communication during AE is lacking.

To characterize the longitudinal evolution of CD8^+^ T-cell states and the spatial distribution of DC–T cell interactions during AE progression, we established a secondary AE infection model in mice and performed integrated single-cell RNA sequencing (scRNA-seq), flow cytometry, and multiplex immunofluorescence imaging across early (3 dpi) and chronic (3 mpi) stages of infection. By mapping lineage composition, transcriptional programs, and spatial interactions, we aimed to delineate the dynamic remodeling of hepatic T-cell immunity during AE, with a particular focus on how chronic antigenic stimulation drives the diversification and functional reprogramming of CD8^+^ T-cell subsets.

## 2 Materials and methods

### 2.1 Animal model and experimental design

Female C57BL/6 mice aged 6–8 weeks were obtained from the Laboratory Animal Center of Ningxia Medical University and maintained under specific pathogen-free (SPF) conditions (22 ± 2 °C, 12-h light/dark cycle) with ad libitum access to food and water. Two independent experimental cohorts were generated using a completely randomized design. The first cohort (n = 3 per group) was used for single-cell RNA sequencing and included an uninfected PBS-injected control group (baseline control), a 3-day post-infection group (3 dpi), and a 3-month post-infection group (3 mpi), representing acute and chronic stages of AE, respectively. The second cohort (n = 5 per group) followed the same grouping strategy and was used for flow cytometry and histological analyses.

Protoscoleces (PSCs) were isolated from laboratory-maintained E. multilocularis-infected gerbils. Harvested cyst material was minced and homogenized in sterile phosphate-buffered saline (PBS), and the homogenate was passed through a 180 μm cell strainer. The filtrate was washed three times by gravity sedimentation (10 min each, at room temperature), during which the supernatant was carefully discarded and the pellet was retained, in 40 mL sterile PBS to remove tissue debris. The recovered PSCs were then digested in 1% pepsin solution (pH 2.0) at a 1:10 (PSC suspension:pepsin, v/v) ratio for 20 min at 37 °C with gentle agitation on a rocking platform. After digestion, the suspension was washed again by gravity sedimentation (10 min, room temperature; supernatant discarded and pellet retained) in 20 mL sterile PBS and passed through a 70 μm cell strainer to remove calcareous corpuscles. PSC viability was evaluated by eosin exclusion and confirmed to be >90%. For experimental infection, mice were inoculated via the portal vein with 1,000 viable PSCs resuspended in 100 μL PBS; uninfected controls received an equal volume (100 μL) PBS via the same route. All procedures were approved by the Institutional Animal Care and Use Committee of Ningxia Medical University (IACUC-2025105).

### 2.2 Tissue processing and preparation of single-cell suspensions

At the designated time points, mice were deeply anesthetized via isoflurane inhalation and subsequently euthanized by cervical dislocation. Following euthanasia, the liver and spleen were promptly removed and weighed to calculate organ indices (organ weight/body weight). A portion of the right hepatic lobe was fixed in 4% paraformaldehyde for histological analysis. The remaining liver tissue was minced and digested using the gentleMACS™ Liver Dissociation Kit (Miltenyi Biotec, Germany) on a DSC-400 tissue dissociator, which combines standardized enzymatic and mechanical dissociation. The resulting cell suspension was passed through a 70 µm cell strainer and further processed with the Debris Removal Solution (Miltenyi Biotec, Germany) by density gradient centrifugation at 4 °C to remove cell debris and non-cellular contaminants. After debris removal, a mixed single-cell suspension containing immune and non-immune hepatic cell populations was obtained. Cell viability was assessed by trypan blue exclusion and exceeded 85% for all samples used for scRNA-seq.

### 2.3 Single-cell RNA sequencing and data analysis

Single-cell suspensions were prepared from liver tissues of mice in the first cohort (n = 3 per group). Single-cell capture, barcoding, and library preparation were performed using the MobiCube^®^ High-Throughput Single-Cell 3′ Transcriptome Kit (v2.0) and the MobiNova^®^-100 system (Mojomics, China). Briefly, single-cell suspensions, barcoded beads, and reagents were loaded onto microfluidic chips, where water-in-oil droplets were generated to achieve single-cell partitioning and mRNA capture. After demulsification, reverse transcription was carried out to generate cDNA containing cell barcodes and unique molecular identifiers (UMIs). The cDNA was then pre-amplified and purified using SPRIselect magnetic beads (Beckman Coulter). Quantification and quality assessment were performed using Qubit 4.0 and the Agilent 2100 Bioanalyzer, ensuring a fragment size distribution of 0.5–2 kb. Qualified samples underwent enzymatic fragmentation, end repair, adaptor ligation, and indexed PCR to construct sequencing libraries. Final libraries were sequenced on an Illumina NovaSeq 6000 (paired-end 150 bp) with a target depth of approximately 50,000 reads per cell. Detailed scRNA-seq quality-control metrics are provided in **Supplementary Data 1**.

Raw FASTQ files were processed using the MobiVision software suite for adapter trimming, sample demultiplexing, barcode and UMI identification, and alignment to the mouse reference genome (mm10), resulting in gene-by-cell expression matrices. Across qualified libraries, the primary processing pipeline identified an estimated total of 78,855 cell barcodes. One 3-mpi library yielded only 565 estimated cells and did not meet sequencing quality requirements; therefore, downstream analyses included samples from the control (n = 3), 3-dpi (n = 3), and 3-mpi (n = 2) groups. Subsequent analyses were performed in R (v4.2) using the Seurat package (v4.0). Cells with <200 or >6,000 detected genes or >10% mitochondrial transcripts were removed. Data were normalized using the LogNormalize method, followed by principal component analysis (PCA) and nonlinear dimensionality reduction with UMAP and t-SNE for clustering. Cell clusters were annotated manually based on canonical marker genes. Differentially expressed genes (DEGs) were identified using the FindMarkers function in Seurat with thresholds of |log_2_FC| > 0.25 and adjusted p < 0.05. Genes displayed in violin plots were selected from DEGs additionally filtered using the following thresholds: |log_2_FC| > 0.25, p_adj < 0.05, pct.1 > 0.25, and pct.2 > 0.25. Enriched Gene Ontology (GO) terms and KEGG pathways were analyzed using the clusterProfiler package to explore immunological functions and signaling pathways associated with AE infection. Full DEG lists, GO/KEGG enrichment results, and cell–cell interaction analysis tables are provided in **Supplementary Data 2**.

### 2.4 Histological and biochemical analyses

Fixed liver tissues were embedded in paraffin and sectioned at 4 µm. After deparaffinization and rehydration, sections were stained with hematoxylin and eosin (H&E) and scanned using an Olympus VS200 digital slide scanner. Pathological features, including inflammatory infiltration, necrosis, and fibrosis, were assessed according to the criteria in **Supplementary Table 1**.

Blood samples were allowed to clot at room temperature and then centrifuged at 3,000 rpm for 10 min; serum ALT, AST, ALP, total bilirubin (TBIL), total protein (TP), and albumin (ALB) were measured using a Chemray 240 automated analyzer (Rayto, China).

### 2.5 Flow cytometry

Single-cell suspensions were prepared from liver tissues as described in Section 2.2, and lymphocytes were enriched by 30%/70% Percoll density-gradient centrifugation(450 × g, 20 min, room temperature; acceleration 1, deceleration 0). Cells were stained with Fixable Viability Stain 510 (BD Horizon, 564406) to exclude dead cells. After Fc receptor blockade with anti-mouse CD16/32 (eBioscience; cat. no. C001T03F01), 1×10^6^ cells per sample for liver and 2×10^6^ cells per sample for spleen were incubated with fluorochrome-conjugated antibody panels targeting immune cell lineage markers and functional markers. Antibody information and dilution ratios are provided in **Supplementary Table 2**. Surface staining was performed for 30 min at 4 °C in the dark. For phenotypic analysis, cells were stained without ex vivo stimulation. For intracellular cytokine analysis, cells were stimulated *in vitro* with protoscolex crude antigen (15 μg/mL) plus anti-CD28 (BioLegend, San Diego, CA, USA; 1 μg/mL) for a total of 18 h at 37 °C and 5% CO_2_. BD GolgiPlug™ Protein Transport Inhibitor (BD Biosciences, San Jose, CA, USA; 1 μL/100 μL) was added during the final 6 h to inhibit protein transport and promote intracellular cytokine accumulation. PMA/ionomycin cell stimulation cocktail (YaMei Biotechnology, Shanghai, China; 2 μL/100 μL) was included as a positive control. After stimulation, cells were harvested and stained for surface lineage markers, followed by fixation for 10 min and permeabilization according to the manufacturer’s instructions. Intracellular staining was then performed for 30 min at 4 °C using the Foxp3/Transcription Factor Staining Buffer Set (BD Biosciences, San Jose, CA, USA). Samples were acquired on a Cytek Aurora full-spectrum flow cytometer. Unstained, single-stained, and fluorescence-minus-one (FMO) controls were included for compensation and gating. Data were analyzed using FlowJo software (v10.8). Gating was performed on singlets and live CD45^+^ cells, followed by lineage-specific markers and functional readouts. Phenotype definitions, including positive and negative markers for each population, are summarized in **Supplementary Table 3**.

### 2.6 Multiplex immunofluorescence staining

Paraffin-embedded liver sections were deparaffinized, rehydrated, and subjected to heat-induced epitope retrieval using EDTA buffer (pH 9.0). Endogenous peroxidase activity was quenched with 3% H_2_O_2_, followed by multiplex fluorescence staining using the AlphaXTSA^®^ multispectral immunohistochemistry kit (Aikefa Biotechnology, Beijing, China). Sections were sequentially incubated with the primary antibodies anti-CD8α (Abcam, ab217344, 1:2000), anti-Granzyme B (Abcam, ab317458, 1:1000), and anti-CD11c (CST, #97585, 1:150), each applied overnight at 4 °C. Signal amplification and fluorophore development were performed according to the manufacturer’s protocol. Nuclei were counterstained with DAPI, and images were acquired under consistent exposure settings using a Zeiss Axio Observer 7 inverted fluorescence microscope.

### 2.7 Statistical analysis

All statistical analyses were performed using GraphPad Prism (v9.0). Data are presented as mean ± SEM. For comparisons of continuous outcomes (e.g., flow-cytometry measurements and serum biochemical parameters) among multiple groups, ordinary one-way ANOVA was used, followed by Tukey’s multiple comparisons test. For scRNA-seq–derived proportional data, each mouse was treated as one biological replicate (one value per mouse). Because group sizes were unequal (3 mpi, n = 2 due to exclusion of one library), Brown–Forsythe and Welch ANOVA tests were applied, followed by Games–Howell’s multiple comparisons test. Statistical significance was defined as p < 0.05. Significance levels are indicated as *p* < 0.05 (*), *p* < 0.01 (**), *p* < 0.001 (***), and *p* < 0.0001 (****); ns, not significant.

## 3 Results

### 3.1 AE infection induces progressive hepatic pathology and immune activation in mice

**Figure 1:**
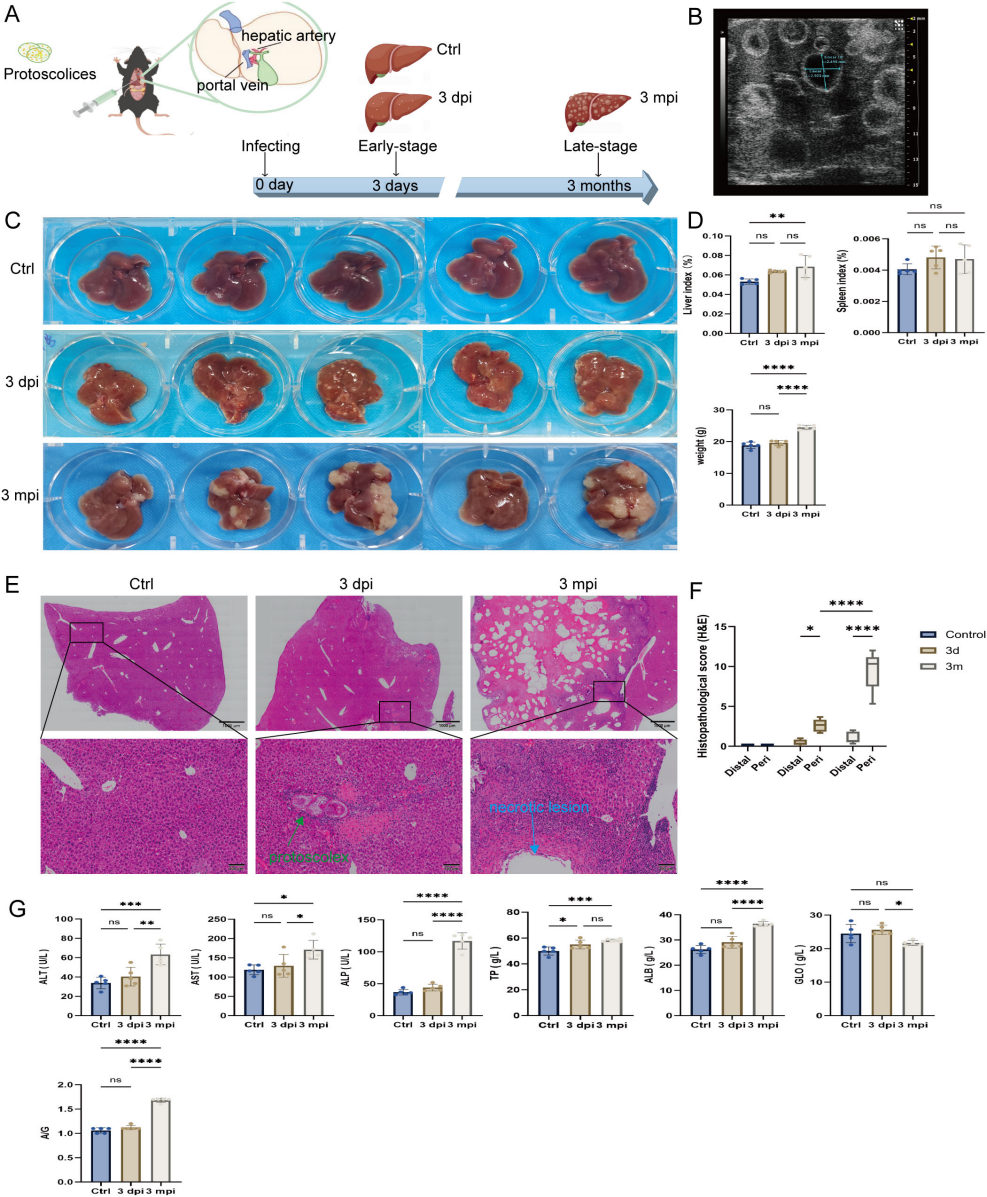
Overview of the AE mouse model and hepatic pathological assessment. **(A)** Schematic illustration of the experimental design showing portal-vein injection of **(E)** multilocularis protoscoleces and the sampling time points at 3 days (3 dpi) and 3 months (3 mpi) post-infection. **(B)** Representative abdominal ultrasound image of a 3 mpi mouse showing multiple hypoechoic nodules in the liver. **(C)** Gross images of livers collected from control, 3 dpi, and 3 mpi mice. **(D)** Liver index, spleen index, and body weight measurements for control, 3 dpi, and 3 mpi groups. **(E)** Representative H&E-stained liver sections from control, 3 dpi, and 3 mpi mice. Lower panels show magnified views of the boxed regions. Green arrow in the 3 dpi section indicates the implanted **(E)** multilocularis protoscolex. Blue arrow in the 3 mpi section indicates a late-stage necrotic lesion. Scale bars: 1000 μm (upper panels) and 100 μm (lower panels). **(F)** Semi-quantitative histopathological scoring based on H&E staining for control, 3 dpi, and 3 mpi mice. **(G)** Serum biochemical parameters (ALT, AST, ALP, TP, ALB, and GLO) in control, 3 dpi, and 3 mpi groups. Data are presented as mean ± SEM. Statistical analyses were performed using one-way ANOVA with appropriate *post-hoc* tests. *p < 0.05, **p < 0.01, ***p < 0.001, ****p < 0.0001; ns, not significant.

To model the temporal course of human AE infection, mice were injected via the portal vein with viable protoscoleces and evaluated across early (3 dpi) and late-stage (3 mpi) infection (**Figure 1A**). At 3 mpi, abdominal ultrasound revealed multiple hypoechoic nodules within the liver parenchyma (**Figure 1B**), consistent with space-occupying lesions. Gross inspection further demonstrated distinct stage-specific changes: scattered punctate necrotic foci were visible on the liver surface at 3 dpi, whereas by 3 mpi the liver was markedly enlarged with extensive infiltrative lesions (**Figure 1C**). The liver index was significantly increased in 3 mpi mice compared with both the uninfected baseline control and 3 dpi groups (*p* < 0.01), while a mild, non-significant increase in spleen index was observed, body weight was also elevated at 3 mpi (**Figure 1D**). These findings indicate successful establishment of the AE infection model and demonstrate progressive hepatic involvement.

Histopathological assessment further delineated the chronological pattern of liver injury (**Figure 1E**). Livers from uninfected baseline control mice exhibited intact lobular architecture. At 3 dpi, acute inflammatory responses were evident around the implantation sites, characterized by dense neutrophil and mononuclear-cell infiltration accompanied by focal hepatocellular necrosis. By 3 mpi, the lesions had evolved into granuloma-like structures with a necrotic core containing parasite remnants, surrounded by epithelioid cells, plasma cells, and lymphocytes, and encapsulated by fibrotic tissue. Normal hepatic architecture was largely replaced by these infiltrative lesions. Semi-quantitative scoring confirmed significantly increased liver injury scores in both 3 dpi and 3 mpi groups relative to uninfected baseline controls (*p* < 0.05), with the highest scores in 3 mpi mice (*p* < 0.01 vs. 3 dpi; **Figure 1F**), reflecting progressive tissue damage throughout infection.

Serum biochemical measurements were consistent with the histological alterations (**Figure 1G**). At 3 mpi, levels of ALT, AST, and ALP were significantly elevated, indicating hepatocellular injury and cholestasis. Mild fluctuations in TP, ALB, and globulin were also detected. In contrast, biochemical markers at 3 dpi remained largely comparable to controls, except for a slight increase in total protein, suggesting that early lesions had not yet progressed to systemic biochemical abnormalities.

Together, these results demonstrate that AE infection induces a continuous progression from acute localized inflammation to extensive chronic hepatic pathology, accompanied by increasing tissue injury and systemic immune activation.

### 3.2 Single-cell RNA sequencing reveals AE-induced remodeling of the hepatic immune landscape

**Figure 2:**
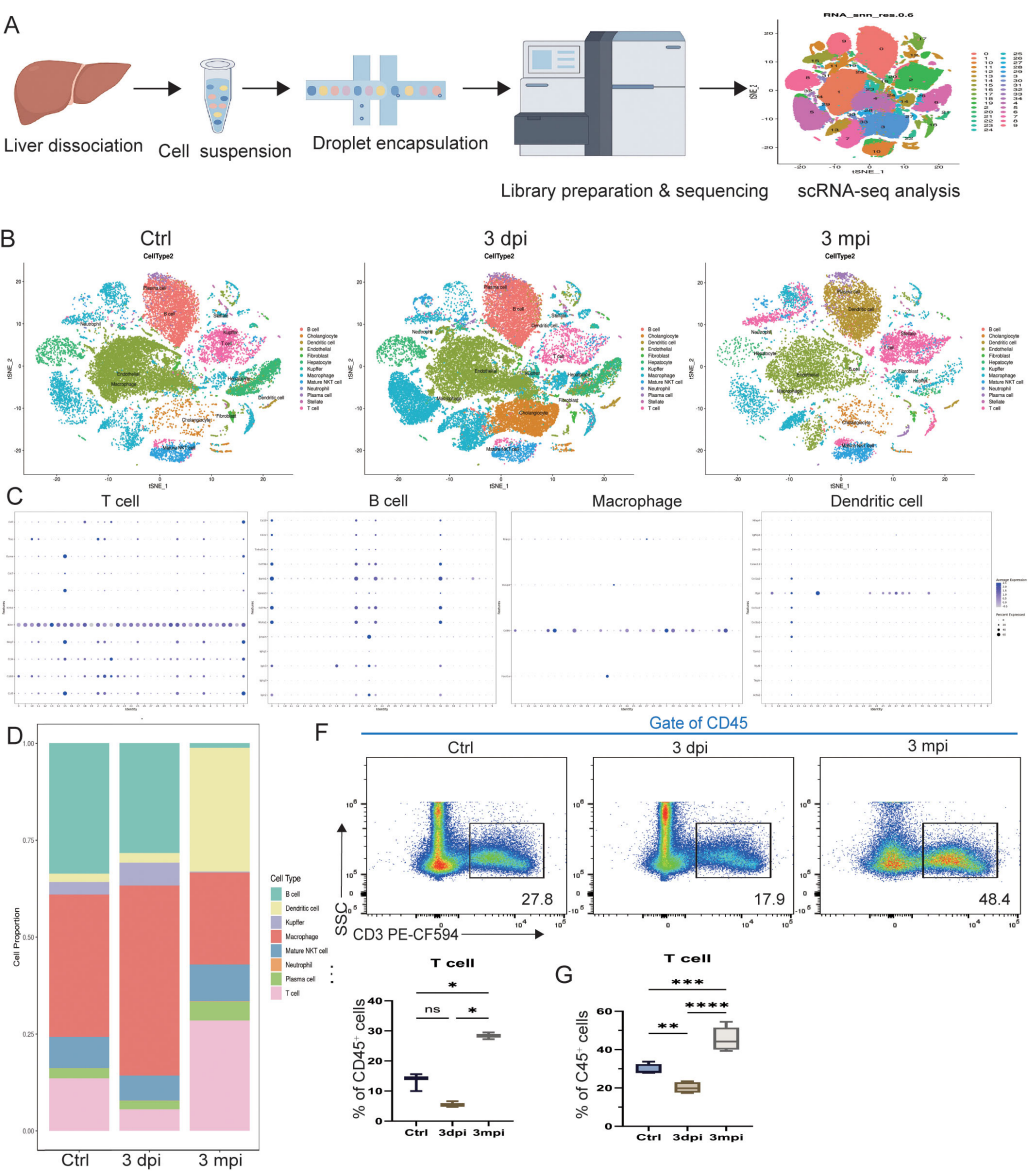
Single-cell RNA sequencing reveals the cellular composition of the hepatic immune microenvironment during AE infection. **(A)** Workflow of single-cell RNA sequencing, including liver tissue dissociation, single-cell capture, library construction, sequencing, and bioinformatic analysis. **(B)** UMAP plots showing major liver cell populations identified in control, 3 dpi, and 3 mpi samples. **(C)** Dot plots displaying canonical marker genes used for annotation of T cells, B cells, macrophages, and dendritic cells. **(D)** Stacked bar plot illustrating the proportional distribution of major immune and non-immune cell types across control, 3 dpi, and 3 mpi groups. **(E)** Quantification of T-cell proportions derived from scRNA-seq analysis. **(F)** Representative flow-cytometry gating strategy for identifying CD45^+^CD3^+^ T cells in control, 3 dpi, and 3 mpi mice. **(G)** Flow-cytometric quantification of CD3^+^ T-cell frequencies among CD45^+^ cells. Data are presented as mean ± SEM. Statistical comparisons were performed using Welch ANOVA. *p < 0.05, **p < 0.01, ***p < 0.001, ****p < 0.0001; ns, not significant.

To characterize the dynamic changes in the hepatic immune microenvironment during AE infection, single-cell RNA sequencing was performed on liver tissues from uninfected baseline control, 3 dpi, and 3 mpi mice (**Figure 2A**). Following primary processing and integration with batch correction, gene-by-cell expression matrices comprising a total of 78,290 cells were obtained, with an average of 1,323 detected genes per cell. Thirteen major cell populations were identified, including T cells, B cells, DCs, macrophages, Kupffer cells, neutrophils, NKT cells, plasma cells, and non-immune populations such as hepatic stellate cells, fibroblasts, hepatocytes, and cholangiocytes. Integrated UMAP visualization revealed marked stage-dependent shifts in cell distribution (**Figure 2B**), and canonical marker expression confirmed accurate annotation (**Figure 2C**).

Focusing on immune compartment dynamics, proportional analysis demonstrated a pronounced temporal reorganization of immune composition during AE infection (**Figure 2D**). In uninfected baseline control mice, macrophages (37%) and B cells (33%) constituted the dominant immune subsets. At 3 dpi, the immune profile shifted toward an acute inflammatory response, characterized by increased macrophages (48%) and reduced B cells (29%) and T cells (6%), indicating reliance on innate immunity at early stages. By 3 mpi, the immune landscape underwent a profound transition, with substantial expansion of T cells (28%) and DCs (32%), accompanied by sharp declines in macrophages (24%) and B cells (1%). Minor fluctuations were also observed in other innate and adaptive subsets.

Given the marked expansion of T cells in the chronic phase and their functional importance, this subset was examined in greater detail. Quantitative scRNA-seq analysis showed a significant increase in T-cell abundance at 3 mpi compared with both uninfected baseline control and 3 dpi groups (**Figure 2E**), suggesting a potential central role for T cells in chronic-stage immune regulation. This pattern was independently validated by flow cytometry, which similarly revealed reduced T-cell proportions at 3 dpi and a significant rebound at 3 mpi (**Figures 2F, G**). Proportional changes in other major immune subsets were likewise confirmed by flow cytometry (**Supplementary Figure S1**), supporting the robustness of the single-cell dataset.

In summary, integrated single-cell transcriptomic and flow cytometric analyses demonstrated a stage-specific reorganization of the hepatic immune landscape during AE infection. The acute phase was characterized by macrophage-dominated inflammatory activity and a transient reduction in adaptive immune cells. In contrast, during the chronic phase, DCs and T cells became the predominant immune populations, indicating a shift from inflammation-driven innate defense toward antigen presentation and T cell–mediated adaptive immunity.

### 3.3 AE infection induces stage-dependent remodeling of cytotoxic, memory, and exhausted CD8^+^ T-cell subsets

**Figure 3:**
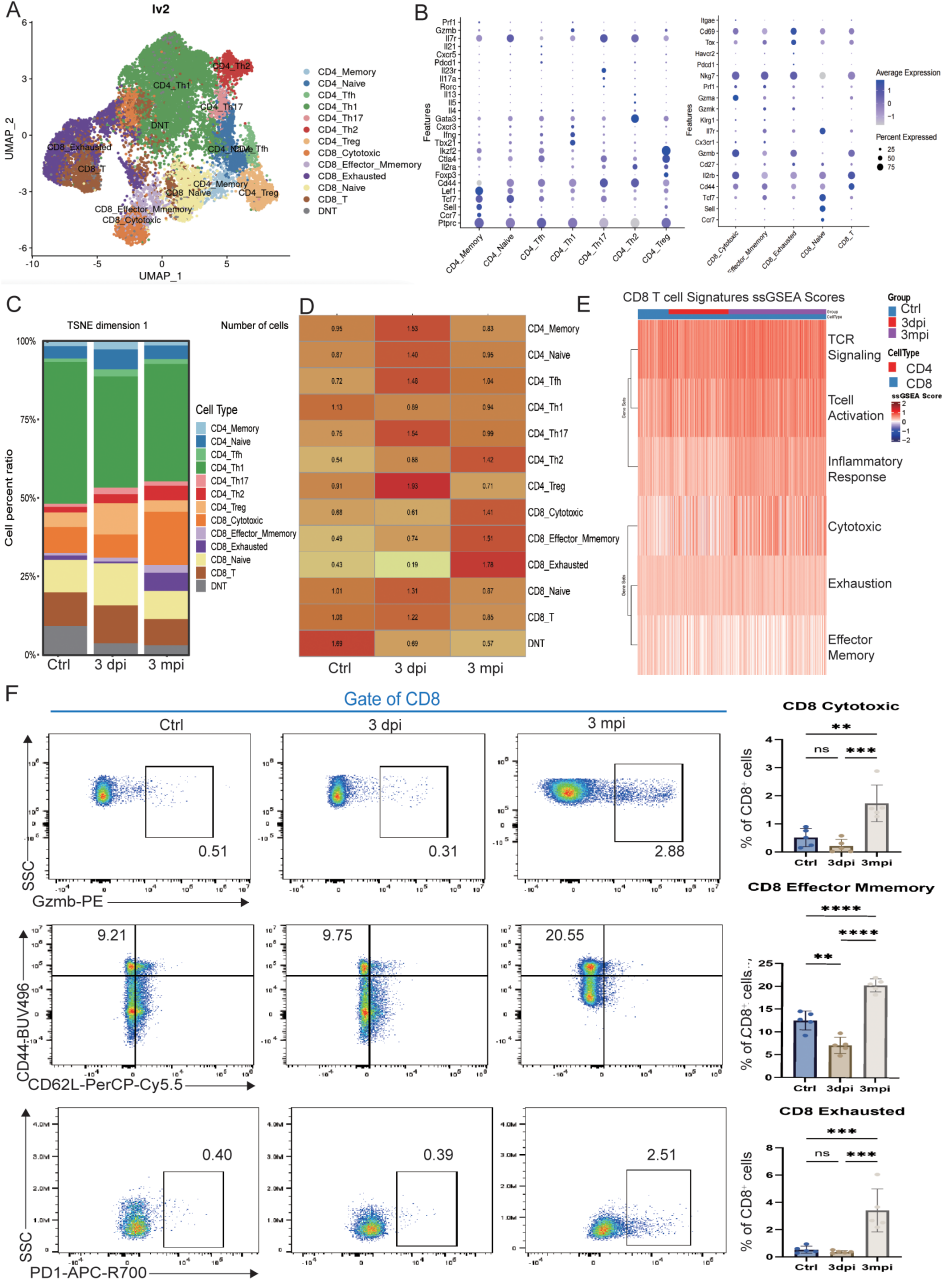
Single-cell and flow-cytometric characterization of T-cell subsets during AE infection. **(A)** UMAP plot of liver-derived T cells showing transcriptionally defined subsets, including CD4^+^ T-cell lineages, CD8^+^ T-cell lineages and DNT cells. **(B)** Dot plots showing canonical marker genes used to annotate each T-cell subset. **(C)** Stacked bar plot displaying the proportional distribution of T-cell subsets (cell percent ratio) in control, 3 dpi, and 3 mpi groups. **(D)** Heatmap showing Ro/e (ratio of observed to expected) values for each T-cell subset across control, 3 dpi, and 3 mpi groups. Ro/e > 1 indicates over-representation of a subset, whereas Ro/e < 1 indicates under-representation relative to expectation. **(E)** Heatmap of ssGSEA scores for CD8^+^ T-cell signatures, including TCR signaling, T-cell activation, inflammatory response, cytotoxic, effector-memory, and exhaustion modules. **(F)** Flow-cytometry analysis of CD8^+^ T-cell subsets. Representative gating of CD8^+^ T cells for Gzmb (CTL), CD44/CD62L (Tem), and PD-1 (Tex) in control, 3 dpi, and 3 mpi mice (left), and corresponding frequencies of CTL, Tem, and Tex among CD8^+^ T cells (right). Data are presented as mean ± SEM. Statistical comparisons for flow-cytometry data were performed using one-way ANOVA with appropriate *post-hoc* tests. **p < 0.01, ***p < 0.001, ****p < 0.0001; ns, not significant.

To delineate the dynamic evolution of T-cell states during AE infection, we performed high-resolution clustering of liver-derived T cells. UMAP analysis identified 13 transcriptionally distinct subsets (**Figure 3A**), including CD4^+^ T-cell lineages (Th1, Th2, Th17, Tfh, Treg, naïve, and memory) and CD8^+^ T-cell lineages consisting of cytotoxic CD8^+^ T cells (CTL), effector-memory CD8^+^ T cells (Tem), exhausted CD8^+^ T cells (Tex), and naïve CD8^+^ T cells, together with a DNT population. Canonical marker expression supported the annotation of each subset (**Figure 3B**).

The distribution of T-cell subsets across groups is visualized in **Figure 3C**, and their relative enrichment is quantified using the Ro/e (ratio of observed to expected) index in **Figure 3D**. At 3 dpi, Th17 (1.54) and Treg cells (1.93) were enriched, reflecting simultaneous induction of pro-inflammatory and regulatory programs. The overall proportion of CD8^+^ T cells remained comparable to controls at this stage. By 3 mpi, however, the T-cell landscape had undergone marked restructuring. CD4^+^ T-cell responses shifted toward a Th2-skewed profile (1.42), whereas CD8^+^ T cells became the predominant adaptive population. Significant enrichment was observed in CTL (1.41), Tem (1.51), and Tex (1.78), indicating the emergence of a diversified cytotoxic–memory–exhaustion configuration during chronic infection. Functional pathway scoring further demonstrated pronounced changes in CD8^+^ T-cell programs at 3 mpi (**Figure 3E**). CD8^+^ T cells exhibited increased enrichment of TCR-signaling, activation, inflammatory, cytotoxic, effector-memory, and exhaustion signatures, reflecting the simultaneous upregulation of effector and inhibitory transcriptional modules under sustained antigen exposure.

Flow cytometry provided quantitative validation of CD8^+^ T-cell subset dynamics (**Figure 3F**). CTL frequencies showed a mild reduction at 3 dpi and higher values at 3 mpi. Tem frequencies were lower at 3 dpi and increased at 3 mpi, whereas Tex frequencies were elevated predominantly at 3 mpi. These flow cytometry-based frequency changes in CTL, Tem, and Tex populations were consistent with the enrichment patterns observed in the scRNA-seq-based Ro/e analysis.

Together, these data demonstrate stage-dependent remodeling of CTL, Tem, and Tex subsets during AE infection, with chronic infection characterized by coordinated changes across cytotoxic, memory-associated, and exhaustion-associated CD8^+^ T-cell programs.

### 3.4 Transcriptomic profiling reveals the molecular basis of CD8^+^ T-cell functional reprogramming

To dissect the molecular processes underlying CD8^+^ T-cell adaptation across AE infection, we performed differential expression and functional enrichment analyses for the CTL, Tem, and Tex subsets. Detailed GO and KEGG enrichment tables with the contributing genes (geneID) for each term are provided in **Supplementary Data 1**.

**Figure 4:**
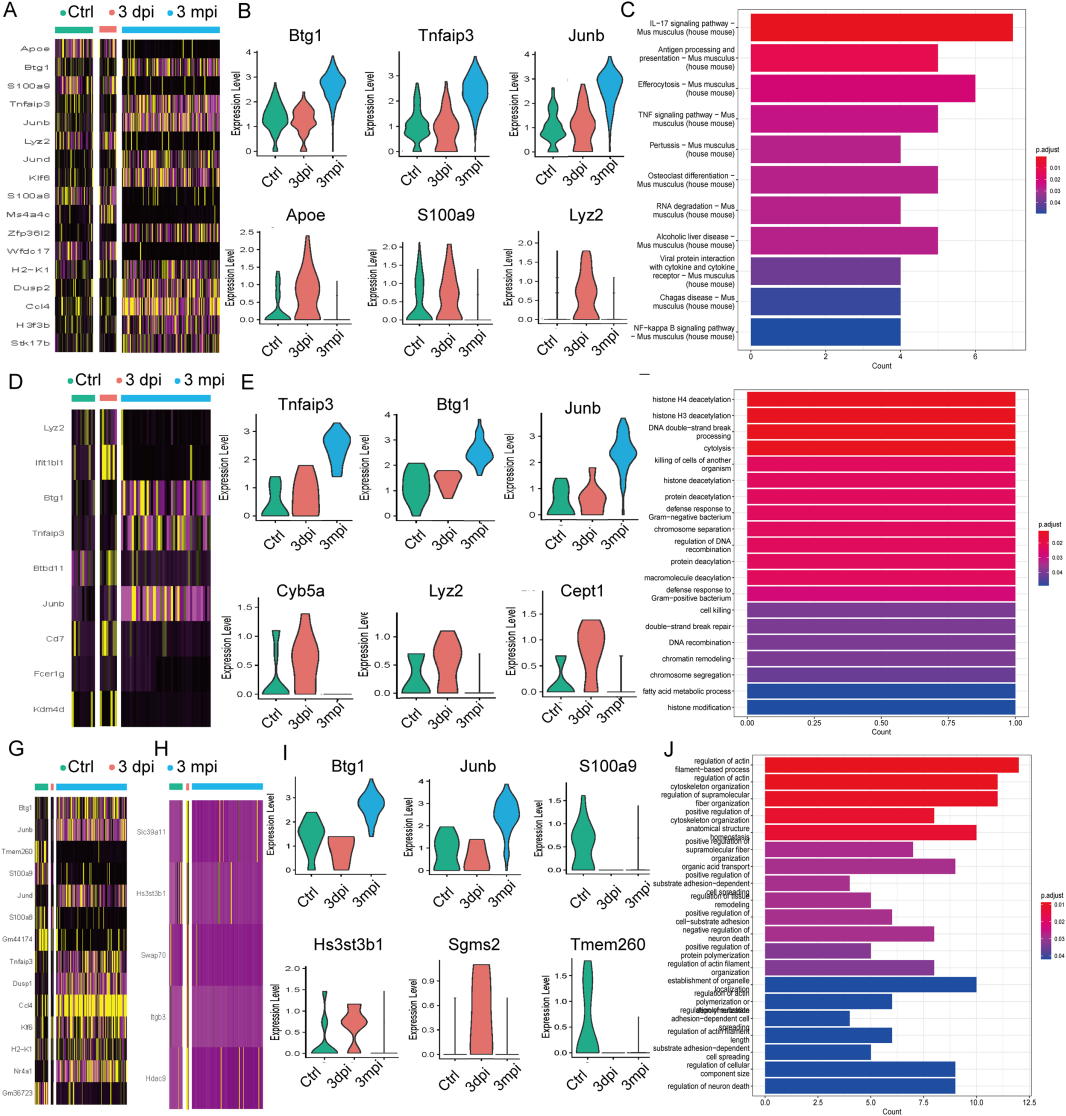
Differential expression and enrichment analyses of CTL, Tem, and Tex subsets across infection stages. **(A)** Heatmap showing DEGs of CTLs from the 3 dpi vs 3 mpi comparison. **(B)** Violin plots showing selected CTL genes from the 3 dpi vs 3 mpi comparison. **(C)** GO enrichment heatmap based on CTL DEGs from the 3 dpi vs 3 mpi comparison. **(D)** Heatmap showing DEGs of Tem cells from the 3 dpi vs 3 mpi comparison. **(E)** Violin plots showing selected Tem genes from the 3 dpi vs 3 mpi comparison. **(F)** GO enrichment heatmap for Tem DEGs from the 3 dpi vs 3 mpi comparison. **(G)** Heatmap showing DEGs of Tex cells from the Ctrl vs 3 mpi comparison. **(H)** Heatmap showing DEGs of Tex cells from the 3 dpi vs 3 mpi comparison. **(I)** Violin plots showing selected Tex genes derived from the Ctrl vs 3 mpi comparison and the 3 dpi vs 3 mpi comparison. **(J)** GO enrichment heatmap for Tex DEGs from the 3 dpi vs 3 mpi comparison.

During early infection (Ctrl vs. 3 dpi), CTLs exhibited robust induction of chemotaxis- and cytoskeleton-associated genes (*Ccl3, Ly6c2, Tmsb4x*), together with upregulation of regulatory and metabolic markers such as *Lgals1* and *Apoe*, indicating rapid recruitment and activation. A subset of signaling-related genes including *Inpp4b* and *Atp8a2* showed early downregulation (**Supplementary Figure S2A**). By the chronic stage (Ctrl vs. 3 mpi), CTLs acquired a distinct transcriptional profile characterized by strong induction of feedback-regulatory transcription factors and *NF-κB* modulators, including *Junb*, *Jund, Btg1, Tnfaip3, Bhlhe40*, and *H3f3b*, accompanied by reduced expression of inflammatory mediators such as *S100a9* (**Supplementary Figure S2B**). Enrichment analysis indicated significant activation of pathways associated with immune regulation, apoptosis-related signaling, and IL-17/TNF-modulated inflammatory circuits (**Supplementary Figure S2C**). Longitudinal comparison (3 dpi vs. 3 mpi) further demonstrated a coherent temporal trajectory: early-induced genes such as *Apoe*, *S100a9*, and *Lyz2* declined over time, whereas chronic-stage regulators *Btg1* and *Tnfaip3* were progressively upregulated (**Figures 4A, B**). GO analysis highlighted sustained involvement of antigen-presentation, efferocytosis, and TNF/IL-17 axis pathways (**Figure 4C**). Together, these patterns suggest that CTLs transition from early migratory and cytotoxic priming toward a more immune-regulatory, feedback-controlled state during chronic infection.

Early after infection (Ctrl vs. 3 dpi), Tem cells exhibited modest activation, including upregulation of *B2m* and *Arap2*, accompanied by reduced expression of *Tmem163* and *Pag1* (**Supplementary Figure S2D**). These features suggest initiation of memory-associated signaling but without large-scale effector activation. At 3 mpi, Tem cells displayed a transcriptional program enriched for negative feedback and immune-regulatory molecules, including *Tnfaip3*, *Btg1*, *Nfkbia*, and *Junb*, together with reduced expression of *Frrs1*, *Fut8*, and *Sh3bgrl3* (**Supplementary Figure S2E**). KEGG analysis revealed enrichment of TNF-associated and regulatory pathways (**Supplementary Figure S2F**). Temporal comparison (3 dpi vs. 3 mpi) revealed downregulation of a set of early-elevated genes (*Cyb5a*, *Lyz2*, *Smarcad1*, *Cept1*, *Ifitbl1*) and increased enrichment of chromatin-regulatory, stress-response, and cytolytic processes (**Figures 4D–F**). These data indicate that Tem cells acquire a low-inflammatory, persistence-oriented transcriptional state during chronic infection, marked by reduced early-phase metabolic activation and strengthened transcriptional control.

In the Tex subset, early infection (Ctrl vs. 3 dpi) induced upregulation of genes involved in cellular stress response and activation (*Slc39a11*, *Plac8*, *Ly6a*) (**Supplementary Figure S2G**). By 3 mpi, Tex cells expressed higher levels of chronic-stimulation markers including *Ccl4*, *Junb*, *Btg*1, and *Nr4a1*, and exhibited reduced expression of inflammatory mediators such as *S100a8/9* and *Tmem260* (**Figures 4G, I**). Enrichment analysis highlighted chemotaxis-related and cytoskeletal-remodeling pathways (**Supplementary Figure S2H**). Longitudinal comparison (3 dpi vs. 3 mpi) revealed progressive upregulation of inhibitory and feedback-regulatory modules (*Btg1*, *Junb*) along with downregulation of early-induced inflammatory genes, and enrichment of pathways related to cell–matrix interactions, cytoskeletal regulation, and adaptive immune signaling (**Figures 4H–J**). These features suggest that Tex cells in chronic AE maintain a hybrid state combining inhibitory signals with persistent activation modules rather than undergoing uniform functional collapse.

In summary, chronic AE infection induces coordinated transcriptional remodeling across CTL, Tem, and Tex subsets. Each subset displays stage-dependent changes involving effector-associated, memory-associated, and inhibitory gene programs. Rather than a unidirectional transition, chronic infection establishes a heterogeneous CD8^+^ T-cell landscape in which cytotoxic, memory, and exhaustion signatures are concurrently enhanced under prolonged antigen exposure.

### 3.5 Spatially organized DC–T cell interaction network in AE lesions

**Figure 5:**
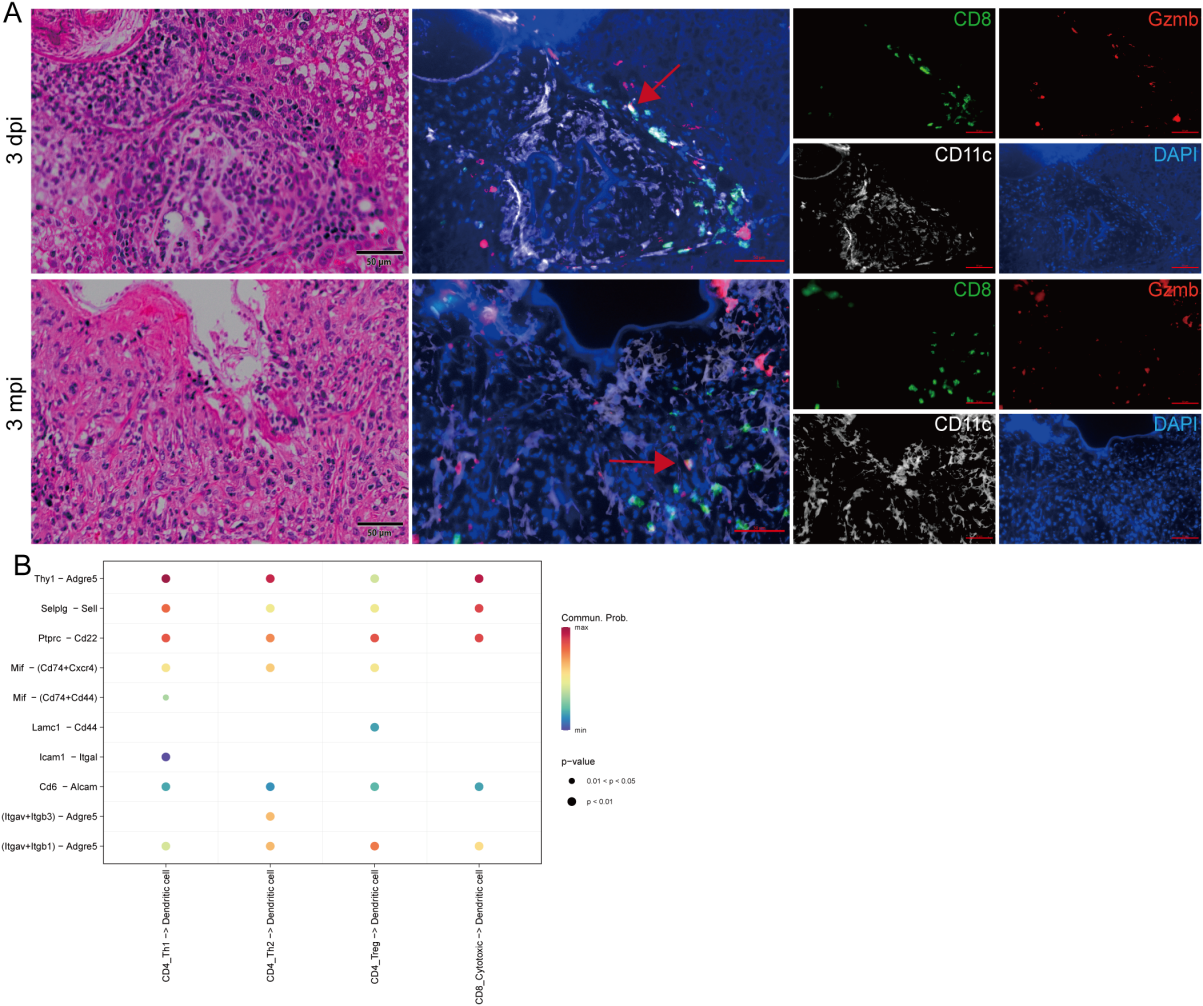
Spatial distribution of DCs and T cells and predicted ligand–receptor interactions. **(A)** Representative histological and multiplex immunofluorescence images of liver sections at 3 dpi and 3 mpi. Left panels: H&E staining. Middle and right panels: multiplex immunofluorescence showing CD11c^+^ dendritic cells (white), CD8^+^ T cells (green), and Gzmb^+^ cells (red), with DAPI nuclear counterstain (blue). Red arrows indicate CTL cells (CD8^+^Gzmb^+^). Scale bars: 50 μm. **(B)** Predicted ligand–receptor interaction matrix between dendritic cells and T-cell subsets derived from single-cell transcriptomic data. Dot color represents communication probability, and dot size indicates statistical significance (*p*-values: *p* < 0.01 or 0.01 ≤ *p* < 0.05).

Multiplex immunofluorescence imaging revealed a consistent spatial organization of immune cells within AE lesions (**Figure 5A**). CD11c^+^ dendritic cells formed dense, circumferential layers surrounding the parasitic structures, while CD8^+^ T cells—including cytotoxic and effector-memory subsets—were distributed immediately adjacent to the DC-rich regions. This arrangement placed CD8^+^ T cells in direct proximity to DCs across both early and chronic stages of infection.

To complement the spatial observations, ligand–receptor prediction based on scRNA-seq data was performed to infer putative DC–T communication within lesions, with a particular focus on cytotoxic CD8^+^ T cells (**Figure 5B**; **Supplementary Data 1**). Among the predicted pairs linking DCs and cytotoxic CD8^+^ T cells, Thy1–Adgre5 showed a prominent interaction signal and met the more stringent significance tier indicated in the plot (p < 0.01). In addition, several adhesion- and trafficking-related axes—such as Selplg–Sell, Icam1–Itgal, and integrin-associated interactions (Itgav+Itgb1/Itgb3–Adgre5)—were also detected, supporting potential mechanisms for DC–CD8 proximity and contact within the lesion niche. Although the prediction framework included CD4^+^ Th1/Th2/Treg subsets as contextual references, the Thy1–Adgre5 axis represented a prominent inferred interaction involving cytotoxic CD8^+^ T cells and DCs (**Figure 5B**).

Integrating spatial and molecular analyses, CD11c^+^ DCs and cytotoxic CD8^+^ T cells exhibited close anatomical localization together with inferred ligand–receptor connectivity, suggesting a spatially adjacent and transcriptionally coupled DC–CD8 interaction landscape within AE lesions.

## 4 Discussion

The liver integrates blood-borne and gut-derived antigens within an immune–metabolic microenvironment that balances activation and tolerance (Zheng and Tian, 2019; Kubes and Jenne, 2018). During AE infection, chronic exposure to parasitic antigens progressively reshapes this environment, leading to coordinated remodeling of innate and adaptive immune populations (Gottstein et al., 2017). By combining single-cell transcriptomics, histopathology, and flow cytometry, our study delineates these dynamic alterations and identifies CD8^+^ T cells as the most prominently reprogrammed lymphocyte population during AE progression.

scRNA-seq revealed that early-stage infection (3 dpi) was characterized by robust innate immune activation. Kupffer cells and infiltrating macrophages dominated the inflammatory response, alongside enhanced Th1-associated signaling. The upregulation of Ifng and enrichment of TCR/NF-κB pathways indicate a rapid pro-inflammatory response to restrict parasite dissemination. This pattern is consistent with classical M1 polarization and IFN-γ/TNF-α–mediated antimicrobial defense (Wang et al., 2020). Further subgroup analysis indicated that early CD8^+^ T-cell responses were dominated by cytotoxic subsets expressing *Gzmb*, *Prf1*, and oxidative phosphorylation–related genes, indicating that CD8^+^ T cells rapidly acquire an inflammatory, metabolically active effector profile. As infection progressed to the late stage (3 mpi), the hepatic immune response shifted from Th1/Th17-associated signatures toward Th2/Treg-oriented regulatory programs (Wang et al., 2018; Vuitton and Gottstein, 2010). This transition reflects a shift toward immune homeostasis and may create a permissive context for subsequent CD8^+^ T-cell diversification. Clinical studies similarly show that AE antigens induce strong and persistent proliferative responses predominantly in CD8^+^ rather than CD4^+^ T cells (Manfras et al., 2002), underscoring the heightened responsiveness of the CD8^+^ lineage to chronic antigenic stimulation.

scRNA-seq and flow cytometry revealed extensive restructuring of intrahepatic CD8^+^ T-cell states at 3 mpi, forming a multi-compartment structure composed of CTL, Tem, and Tex subsets. CTLs retained high levels of *Gzmb* and *Prf1*, Tem cells expressed *Il7r* and *Cx3cr1* consistent with long-term persistence and recall capacity, while Tex cells upregulated inhibitory receptors such as *Pdcd1*, *Lag3*, and *Tox*. Importantly, effector molecules such as *Gzmb* remained detectable within a portion of Tex cells, and the proportion of GZMB^+^ CD8^+^ T cells increased at 3 mpi, indicating that exhaustion in AE is characterized by heterogeneous functional states rather than uniform decline. This heterogeneity resembles the Tpex–Tex hierarchy described in chronic infection and cancer (Beltra et al., 2020; Lan et al., 2023), where progenitor-like exhausted T cells maintain proliferative potential and replenish terminally exhausted cells. *Gzm*b expression associated with non-fertile cysts in cystic echinococcosis, further suggests that cytotoxic activity may contribute to restricting parasite growth while sustaining low-level inflammation (Sweed et al., 2023). Together, these findings indicate that AE infection does not drive a linear collapse of CD8^+^ T-cell function but instead establishes a diversified and balanced CD8^+^ T-cell network in which cytotoxic, memory-like, and inhibitory states coexist under chronic antigen exposure.

At the molecular level, the three CD8^+^ subsets showed both subset-specific features and shared regulatory modules shaped by persistent antigen stimulation. Negative-regulatory molecules such as *Btg1*, *Tnfaip3*, *Junb*, and *Nr4a1* were consistently upregulated across subsets, suggesting reinforced feedback inhibition along the TCR–NF-κB axis and activation-induced stress control (Wherry and Kurachi, 2015). Metabolism-associated genes (*Apoe*, *Inpp4b*, *Atp8a2*) and stress-responsive factors (*S100a9*, *Hspa1a*) were also induced, indicating coordinated metabolic–stress coupling as a mechanism to balance effector activity and maintain cellular fitness. *ApoE*’s known role in membrane organization and T-cell signaling supports the possibility that metabolic remodeling contributes to sustained yet controlled CD8^+^ T-cell activity during chronic AE (Tenger and Zhou, 2003; Bonacina et al., 2018). Collectively, these findings suggest that CD8^+^ T cells undergo self-limiting transcriptional reprogramming, integrating effector, metabolic, and inhibitory pathways to preserve baseline defense while avoiding terminal exhaustion. Notably, this reprogramming was not uniform across CD8^+^ subpopulations. Based on differential expression analyses across stages/groups, the CTL subset exhibited the largest DEG burden and effect sizes in our comparisons, suggesting that CTLs may be particularly responsive to persistent antigen stimulation and microenvironmental changes and thus represent a major contributor to the CD8^+^ T-cell reprogramming observed in this study. In contrast, Tem cells were characterized by higher expression of memory-associated markers, whereas Tex cells showed enrichment of checkpoint/exhaustion-associated signatures, supporting a stratified and heterogeneous state landscape rather than a single linear trajectory.

Spatial analyses reinforce this model. Multiplex immunofluorescence revealed dense circumferential accumulation of CD11c^+^ dendritic cells around parasitic lesions, with CD8^+^ T cells positioned immediately adjacent to these DC-rich regions. This spatial organization suggests that the lesion periphery may function as a structural niche for sustained antigen presentation and T-cell regulation. Ligand–receptor analysis identified several potential DC–T cell communication axes, including Thy1–Adgre5, Mif–Cd74/Cd44, and Icam1–Itgal, which have established roles in adhesion and migratory regulation (Wandel et al., 2012; Gore et al., 2008). While the precise functional consequences of these interactions remain to be defined, their combined spatial and transcriptional features support active DC–T-cell communication in shaping chronic-stage immune equilibrium. Parasite-derived molecules may further modulate this process: hydatid fluid induces DC autophagy and promotes multifunctional T-cell responses, whereas Antigen B suppresses DC maturation and promotes Th2 polarization (Rigano et al., 2007; Chop et al., 2024).

These insights suggest potential immunological intervention strategies in AE. Targeting exhaustion-associated pathways such as *TIM-3* or *TIGIT* has been shown to restore CD8^+^ T-cell functionality (Zhao et al., 2021; Zhang et al., 2020). Modulating metabolic pathways or optimizing mitochondrial fitness may benefit chronically stimulated T cells (Lin et al., 2025). Fine-tuning DC–T-cell communication, particularly along axes such as Thy1–Adgre5 or Mif–Cd74/Cd44, may further enhance local immune responses during chronic AE infection.

This study has several limitations. First, transcriptomic analyses reflect mRNA abundance and cannot fully capture protein-level function or establish causality for DC–T-cell interactions without perturbation-based experiments or spatially resolved approaches. Second, the control group served as an uninfected baseline reference rather than time-matched controls for each time point; therefore, potential time/age-related effects cannot be completely excluded, and baseline comparisons should be interpreted with caution. Third, clonal structure and lineage relationships among CD8^+^ subsets were not addressed and will require TCR-based analyses. Finally, although DC expansion was observed during late-stage AE, DC functional differentiation and its contribution to immune regulation and T-cell activation were not systematically characterized in this study and warrant further investigation.

## 5 Conclusions

In this study, single-cell transcriptomics combined with histopathology, multiplex immunofluorescence, and flow cytometry revealed how Echinococcus multilocularis infection progressively reshapes the hepatic immune landscape. Acute infection induced rapid activation of innate and adaptive responses, whereas chronic infection was marked by the sustained coexistence of cytotoxic, memory-associated, and inhibitory CD8^+^ T-cell programs rather than uniform functional decline. These findings demonstrate that CD8^+^ T cells undergo broad but coordinated transcriptional adjustments under prolonged antigen exposure, forming a diversified compartment composed of cytotoxic, effector-memory, and exhausted subsets. Spatial analyses further showed that CD11c^+^ dendritic cells accumulated around lesion margins in close proximity to CD8^+^ T cells, and ligand–receptor predictions highlighted several candidate interactions, including Thy1–Adgre5, suggesting context-dependent DC–T-cell communication during chronic infection. Together, these results provide an integrated view of T-cell remodeling in AE and offer insights into the immunoregulatory pathways that may shape host–parasite coexistence in the chronic stage.

## Data Availability

The datasets presented in this study can be found in online repositories. The scRNA-seq dataset generated in this study has been deposited in the NCBI repository under the BioProject accession number PRJNA1399072. The corresponding BioSample records have been registered (SAMN54473151–SAMN54473159).
