# Supplementary figures and images for: Alveolar echinococcosis drives functional reprogramming of hepatic CD8+ T cells
Source: Front Cell Infect Microbiol. 2026 Feb 19;16:1747682. doi: 10.3389/fcimb.2026.1747682 (PMC12960575; doi:10.3389/fcimb.2026.1747682)

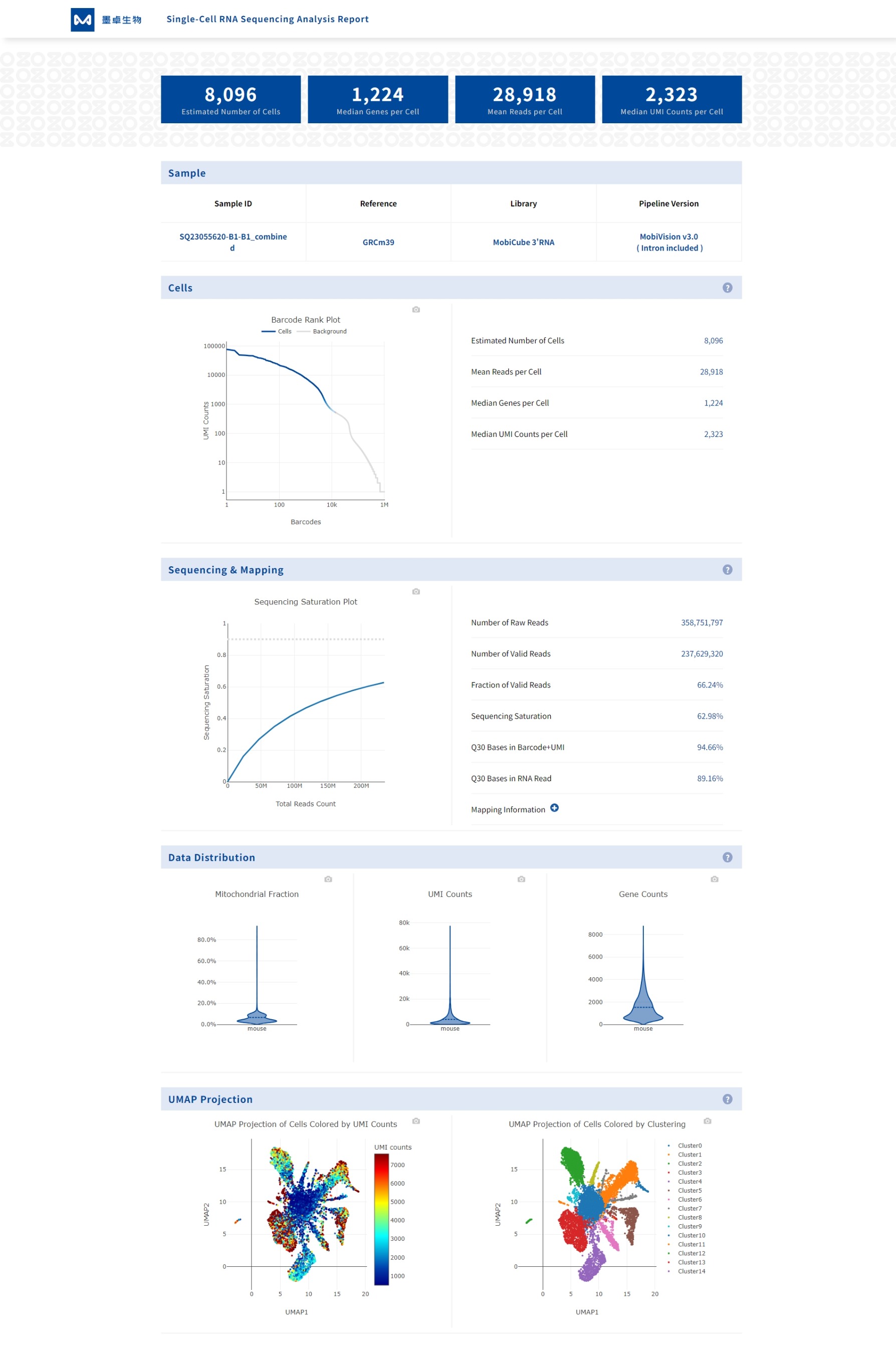

Supplement: Supplementary file 1 [file DataSheet1.zip › Supplementary Data 1/3dpi-B1.jpeg]

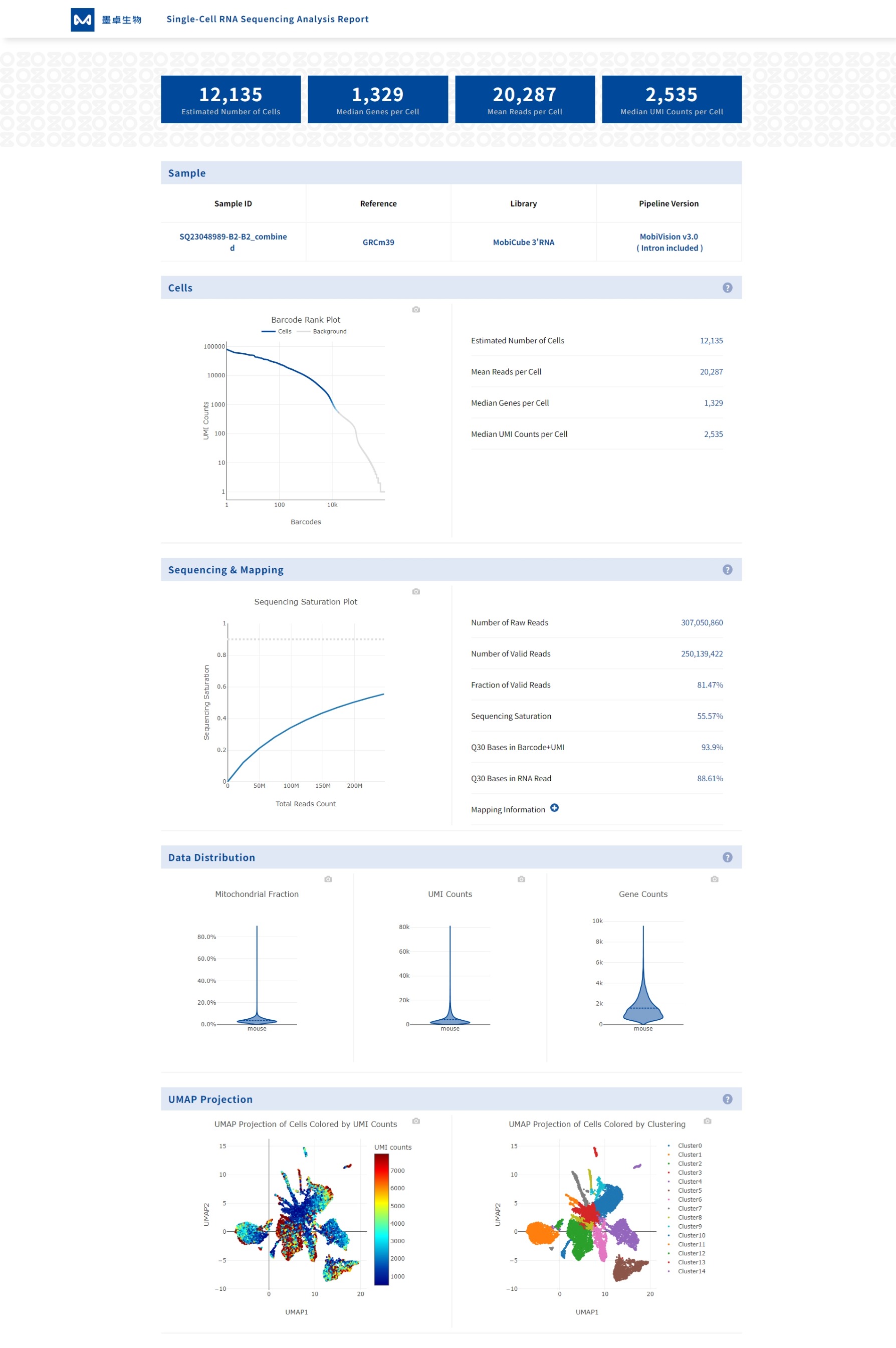

Supplement: Supplementary file 1 [file DataSheet1.zip › Supplementary Data 1/3dpi-B2.jpeg]

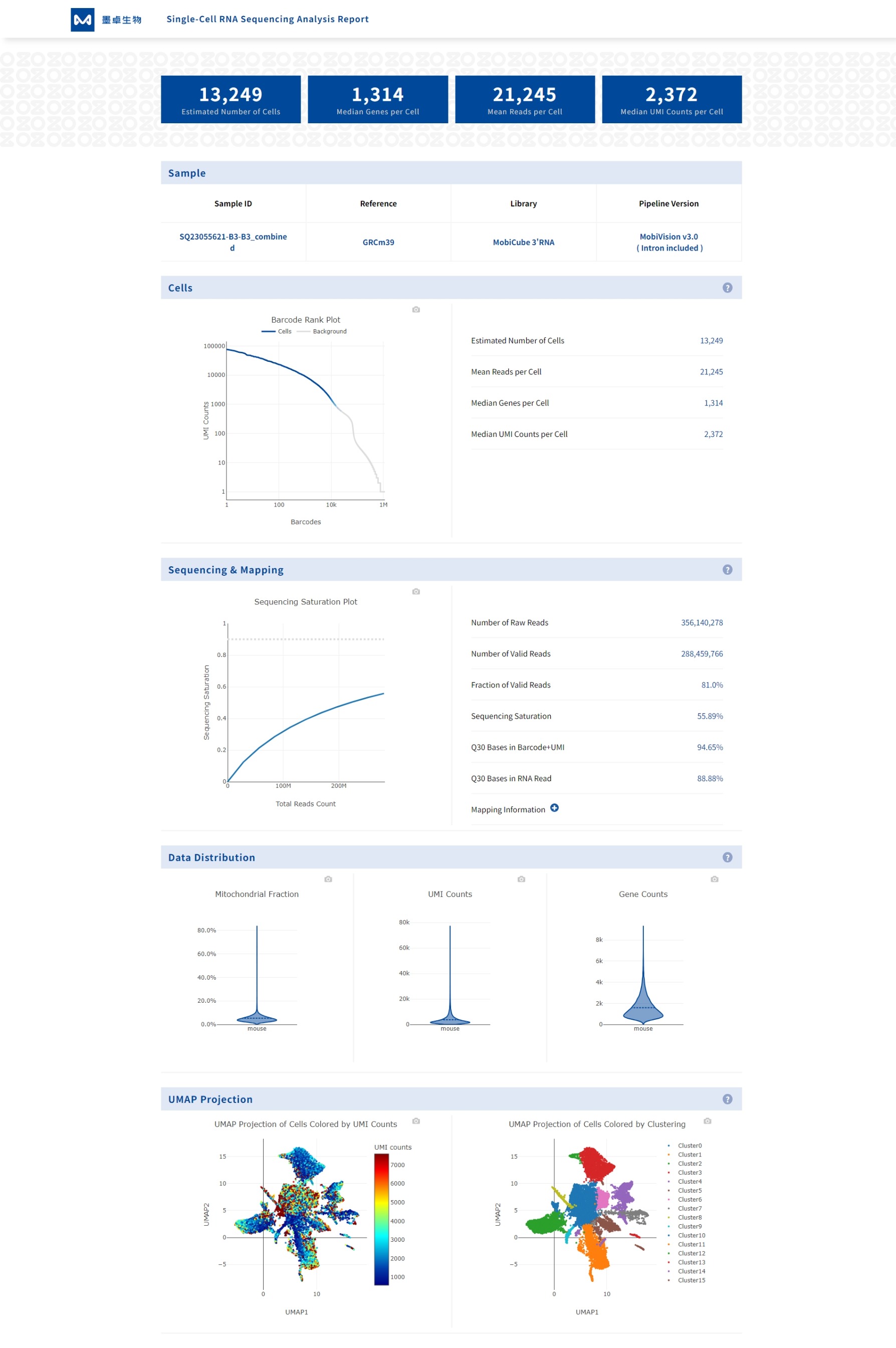

Supplement: Supplementary file 1 [file DataSheet1.zip › Supplementary Data 1/3dpi-B3.jpeg]

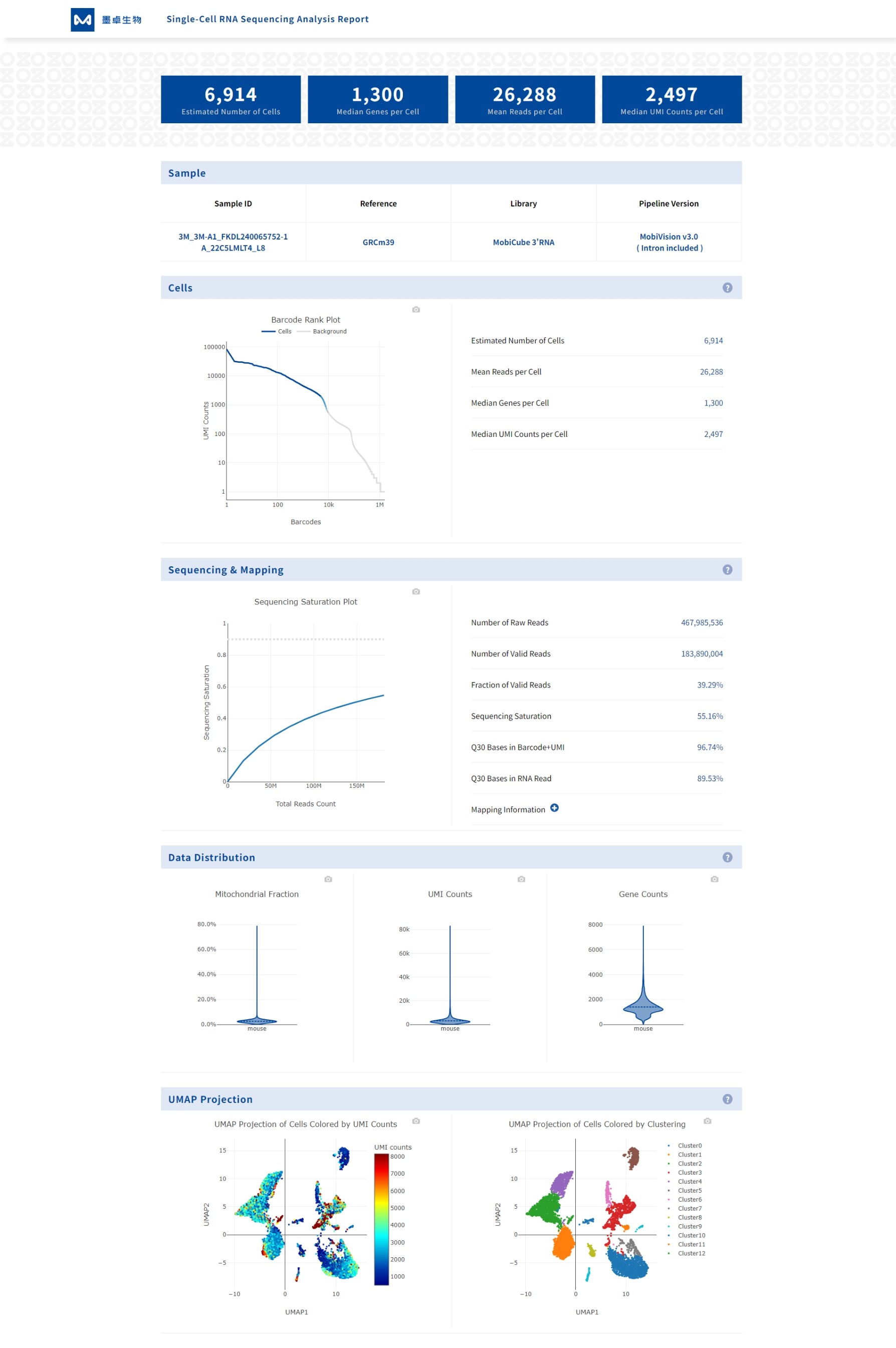

Supplement: Supplementary file 1 [file DataSheet1.zip › Supplementary Data 1/3mpi-C1.jpeg]

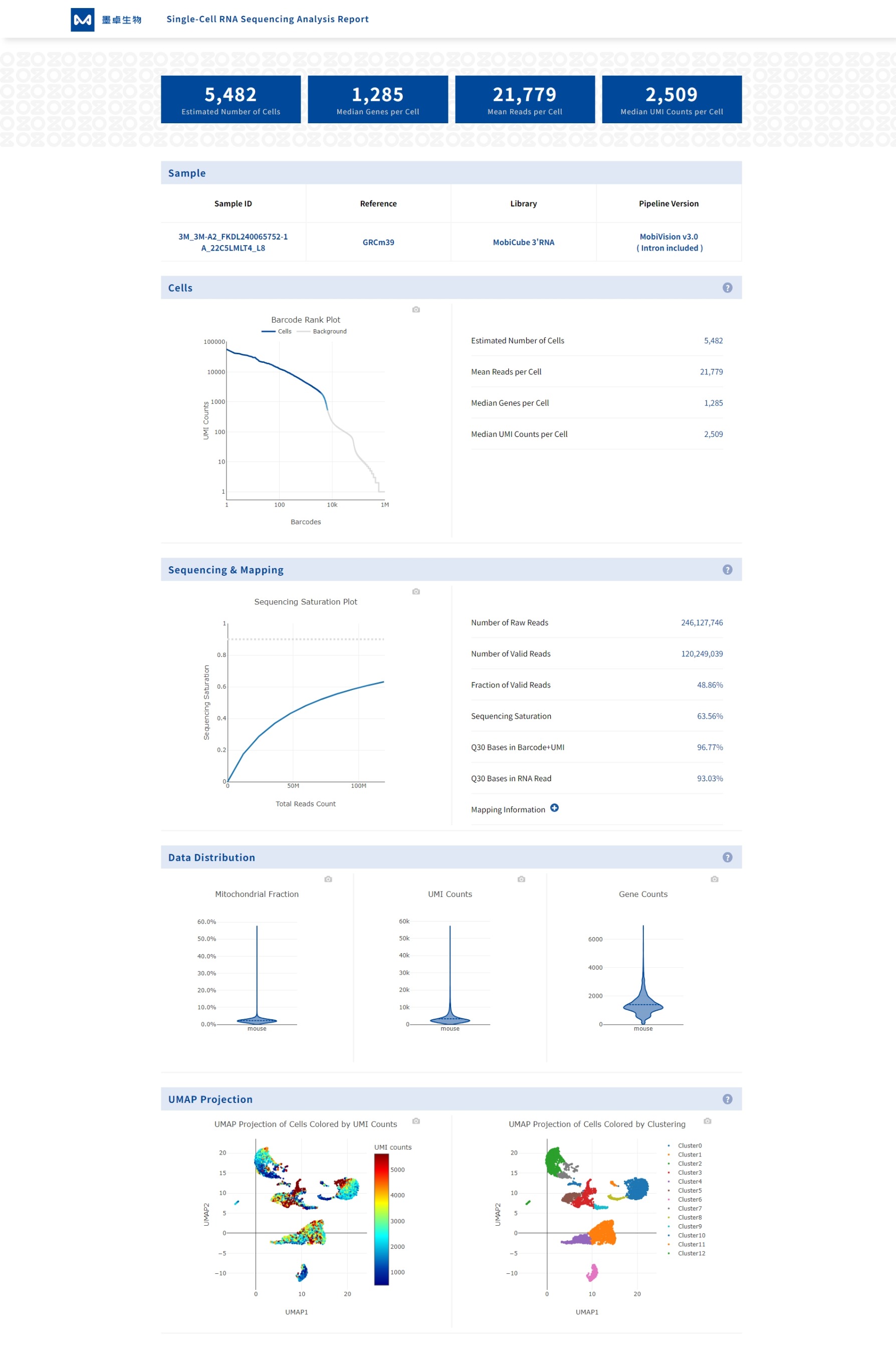

Supplement: Supplementary file 1 [file DataSheet1.zip › Supplementary Data 1/3mpi-C2.jpeg]

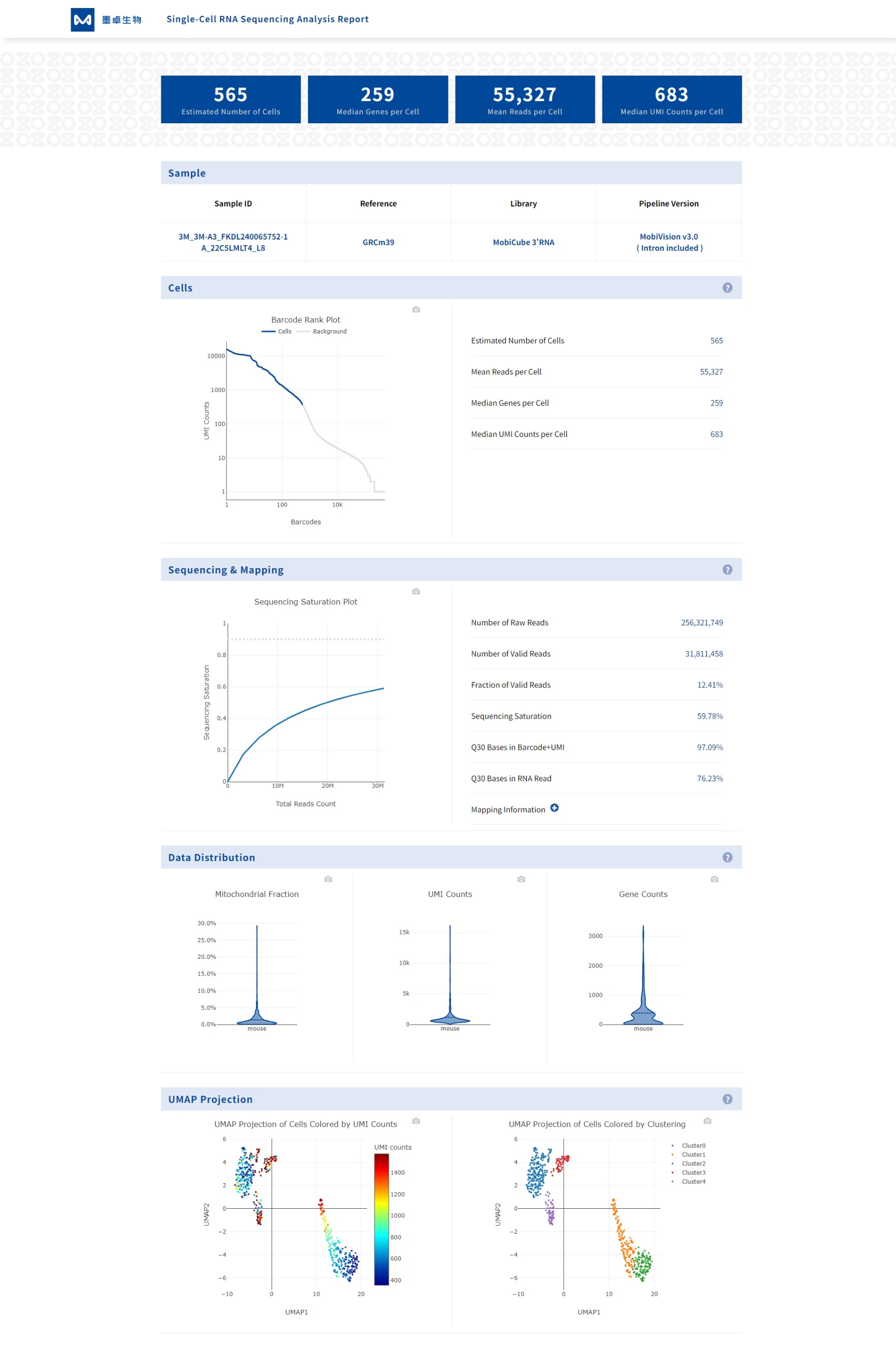

Supplement: Supplementary file 1 [file DataSheet1.zip › Supplementary Data 1/3mpi-C3.jpeg]

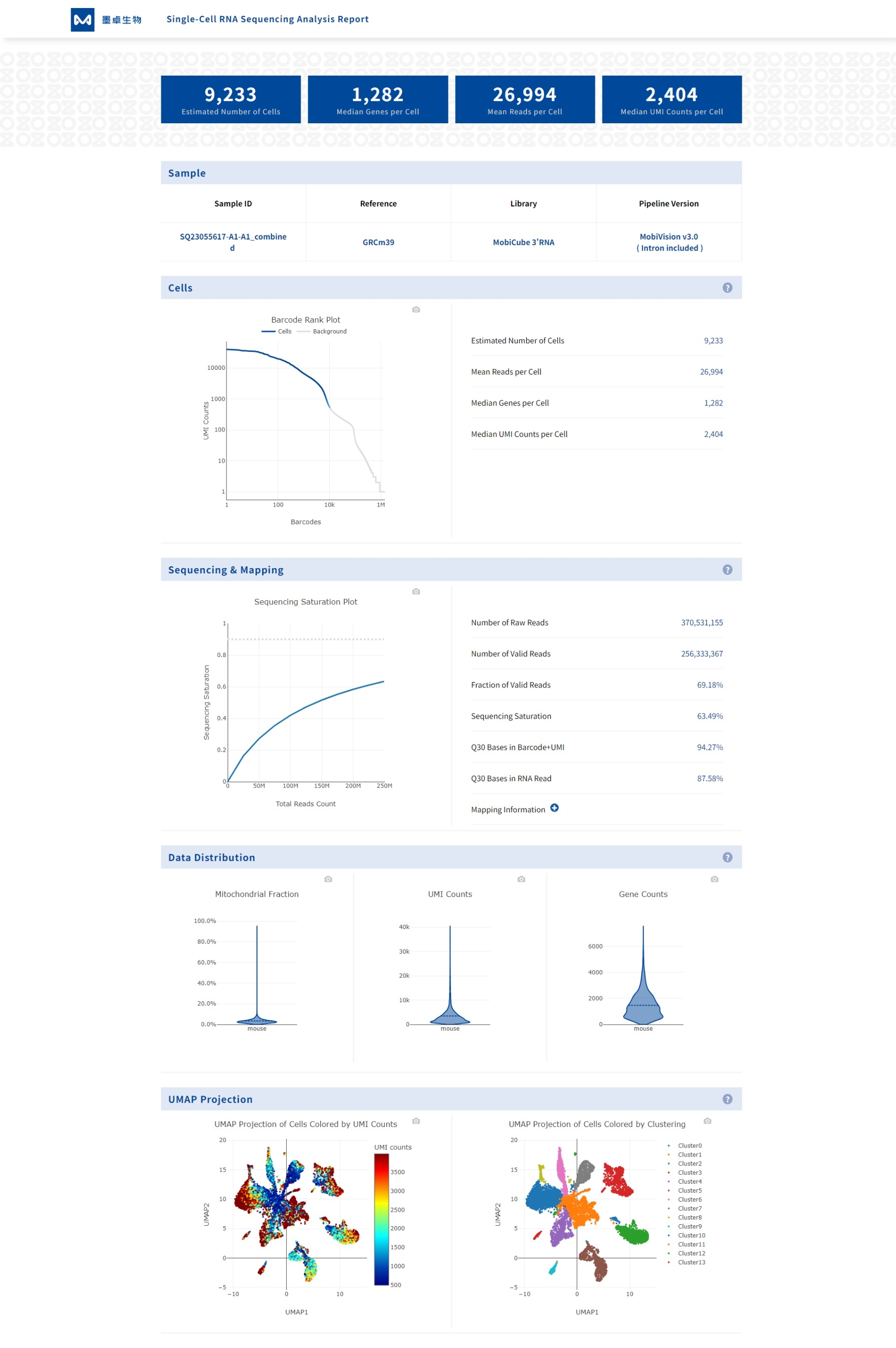

Supplement: Supplementary file 1 [file DataSheet1.zip › Supplementary Data 1/Ctrl--A1.jpeg]

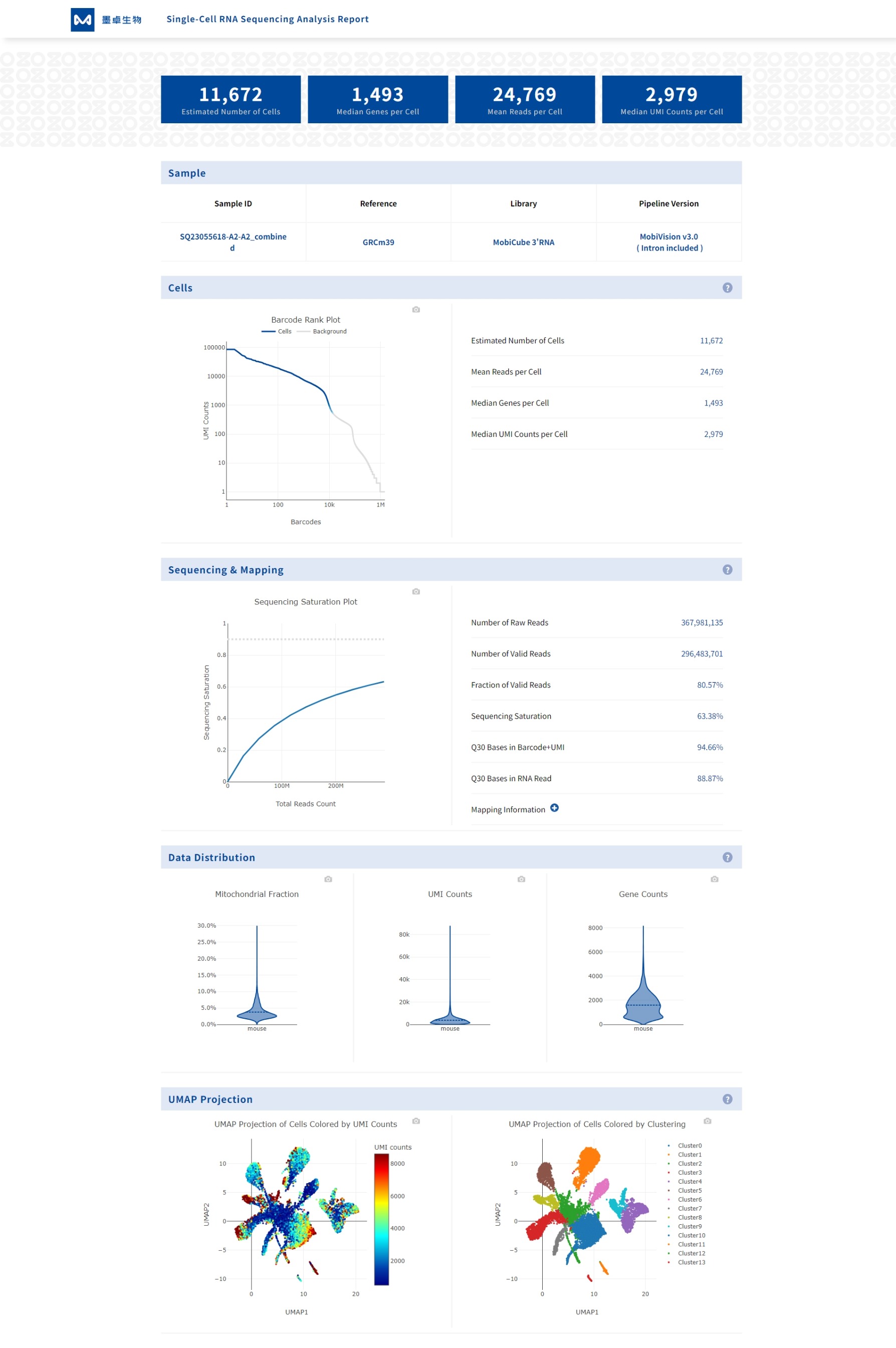

Supplement: Supplementary file 1 [file DataSheet1.zip › Supplementary Data 1/Ctrl--A2.jpeg]

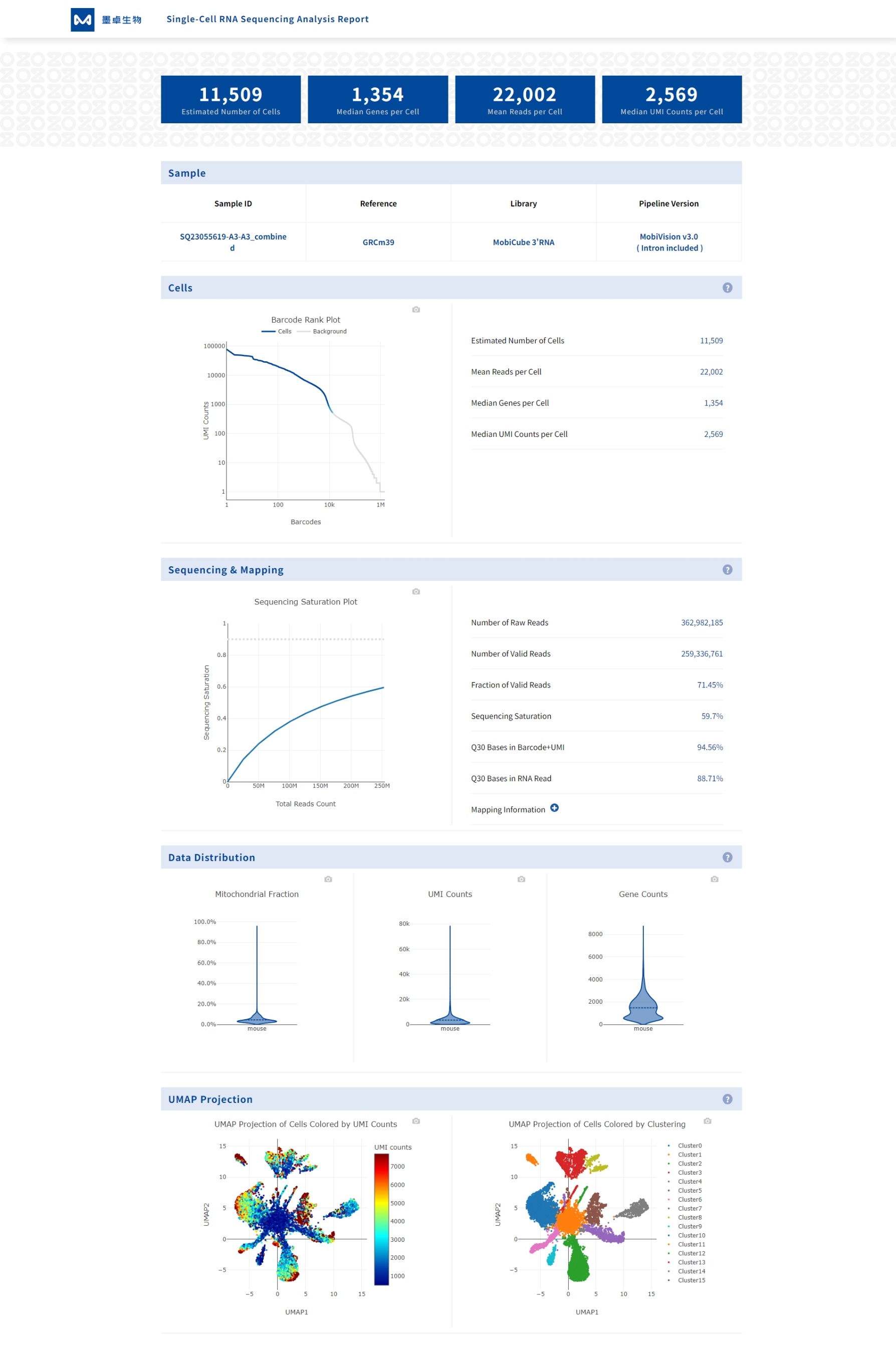

Supplement: Supplementary file 1 [file DataSheet1.zip › Supplementary Data 1/Ctrl--A3.jpeg]

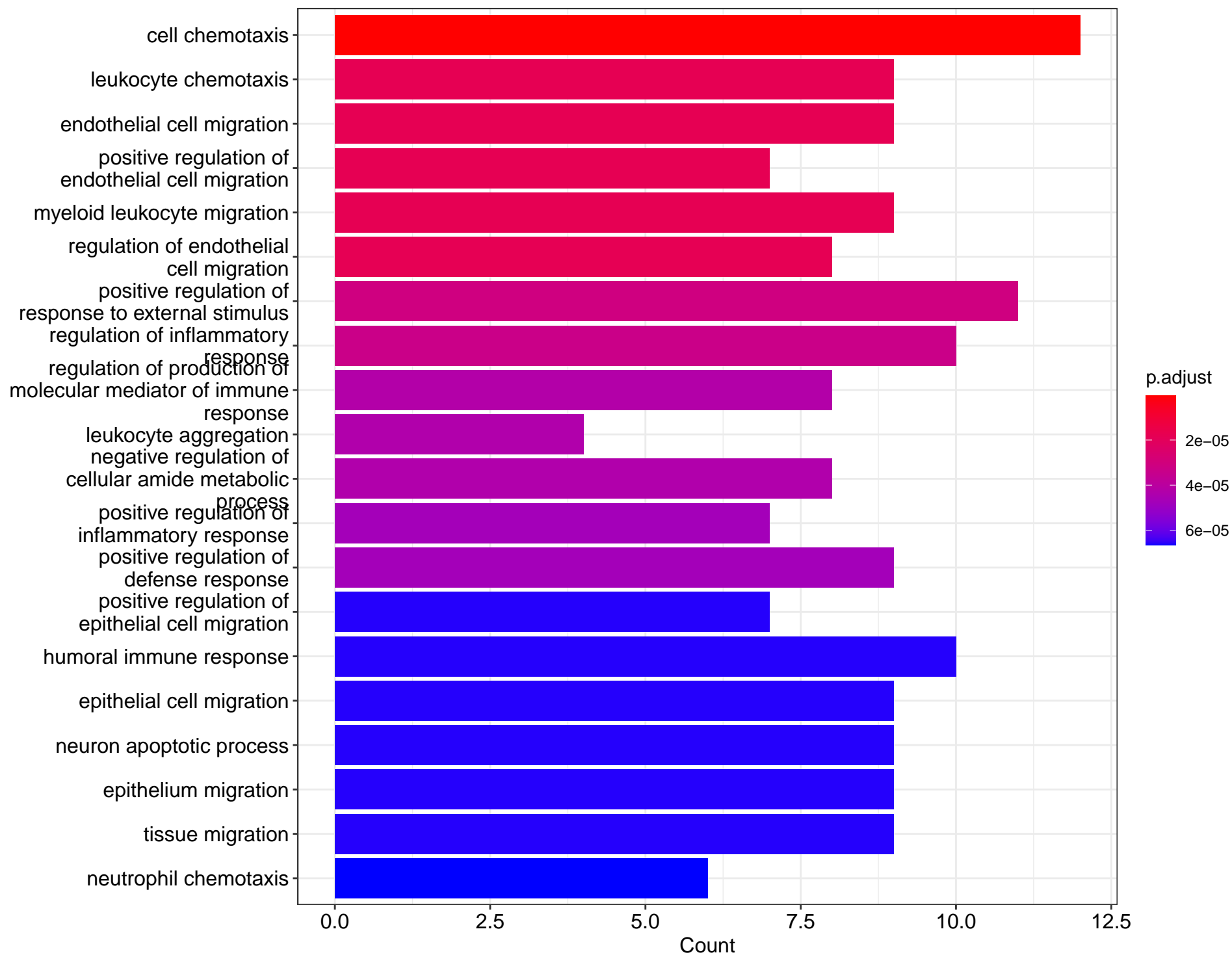

Supplement: Supplementary file 2 [file DataSheet2.zip › Supplementary Date 2/Enrichment_GO_KEGG_with_geneSymbol/CD8_Cytotoxic 3dpi vs 3mpi/3D-A vs 3M-A GO_visualization.pdf]

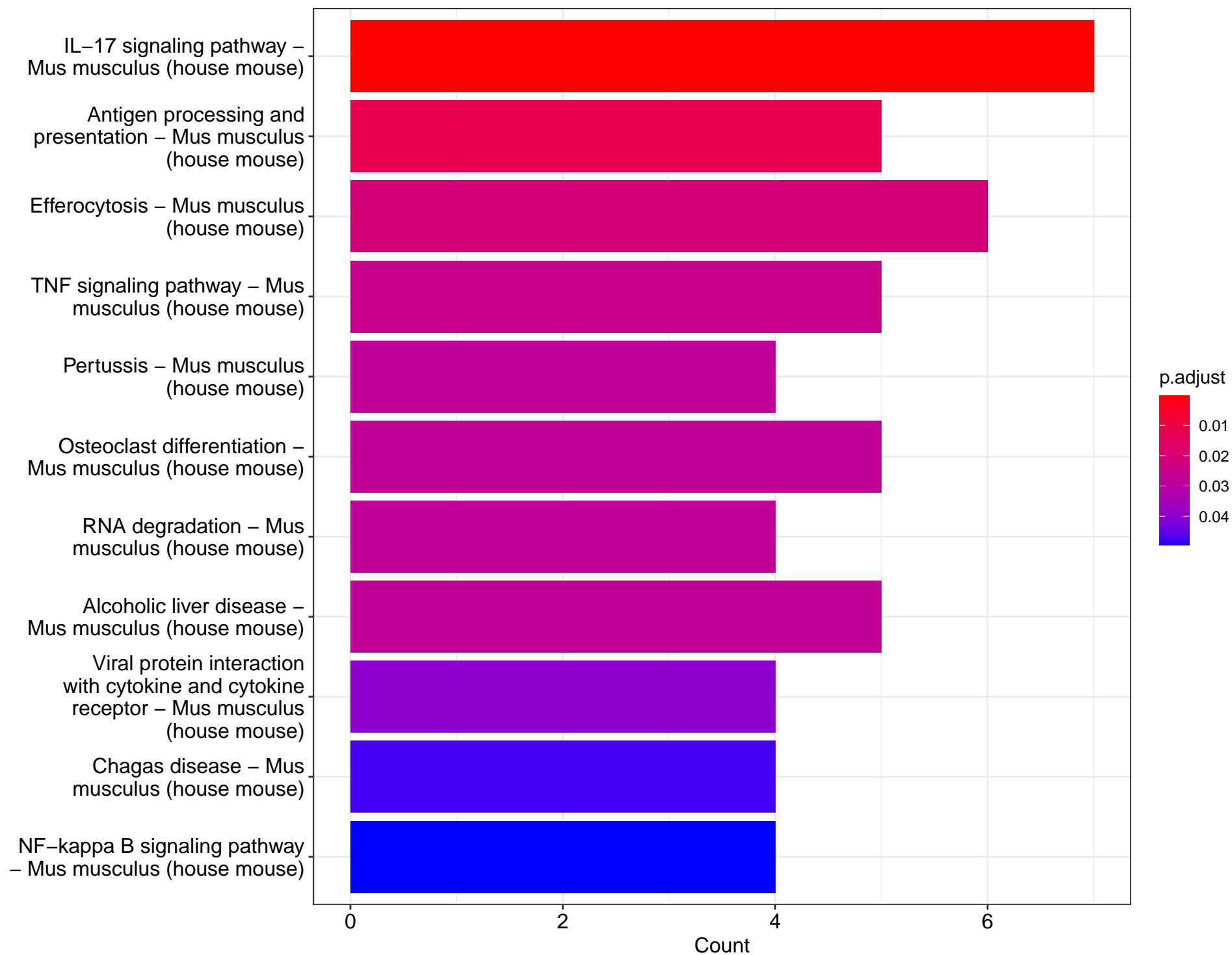

Supplement: Supplementary file 2 [file DataSheet2.zip › Supplementary Date 2/Enrichment_GO_KEGG_with_geneSymbol/CD8_Cytotoxic 3dpi vs 3mpi/3D-A vs 3M-A KEGG_visualization.pdf]

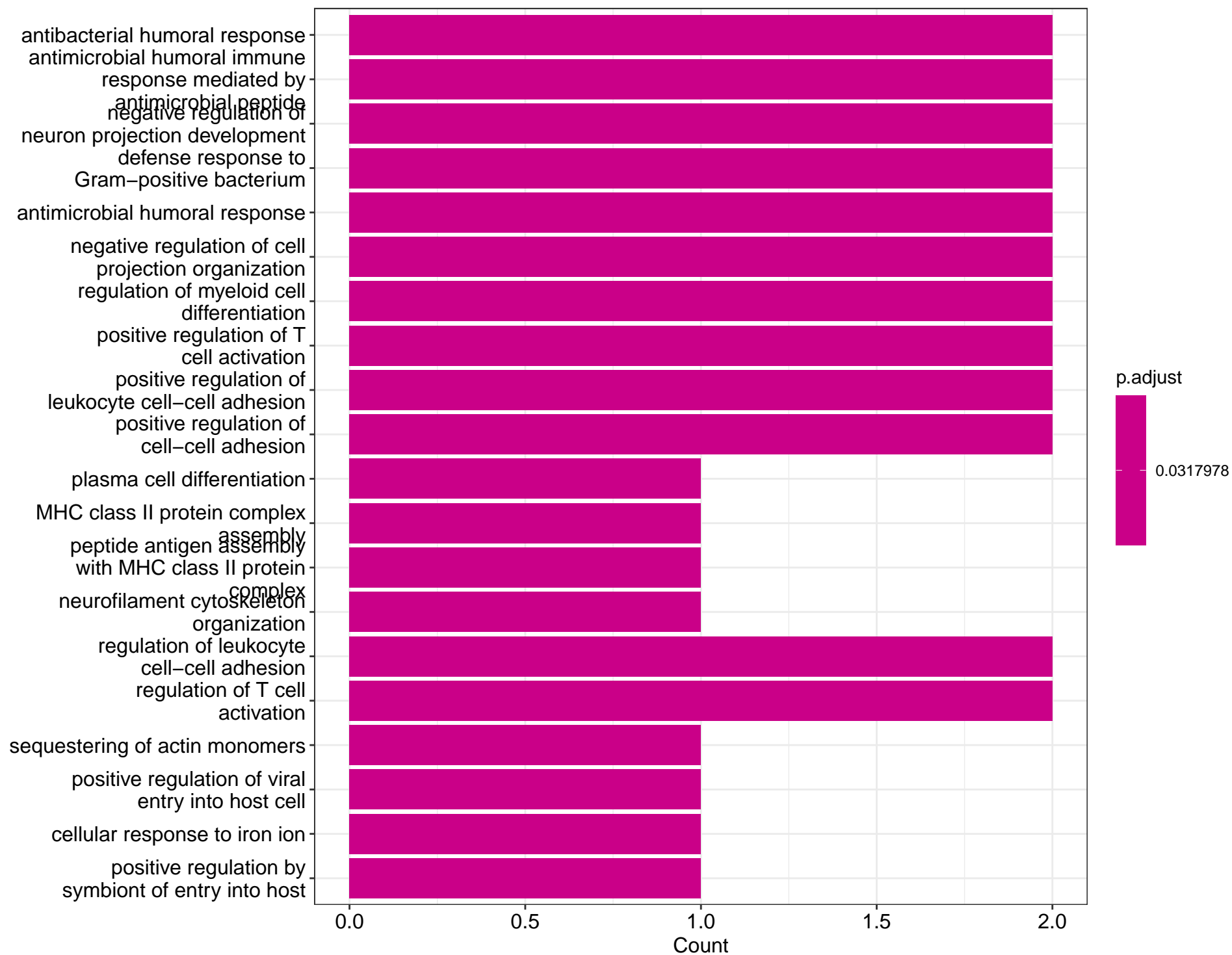

Supplement: Supplementary file 2 [file DataSheet2.zip › Supplementary Date 2/Enrichment_GO_KEGG_with_geneSymbol/CD8_Cytotoxic Ctrl vs 3dpi/3D-PBS vs 3D-A GO_visualization.pdf]

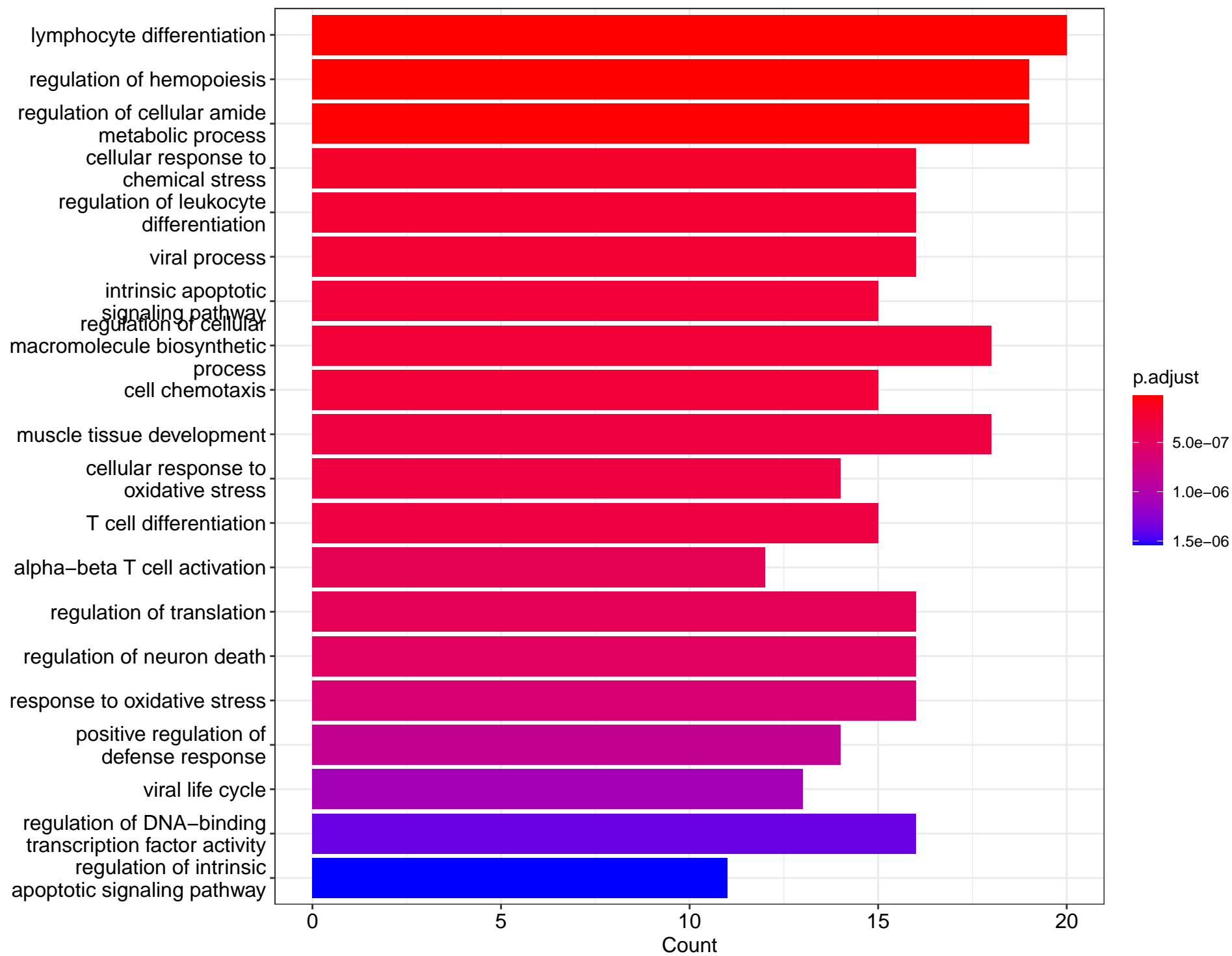

Supplement: Supplementary file 2 [file DataSheet2.zip › Supplementary Date 2/Enrichment_GO_KEGG_with_geneSymbol/CD8_Cytotoxic Ctrl vs 3mpi/3D-PBS vs 3M-A GO_visualization.pdf]

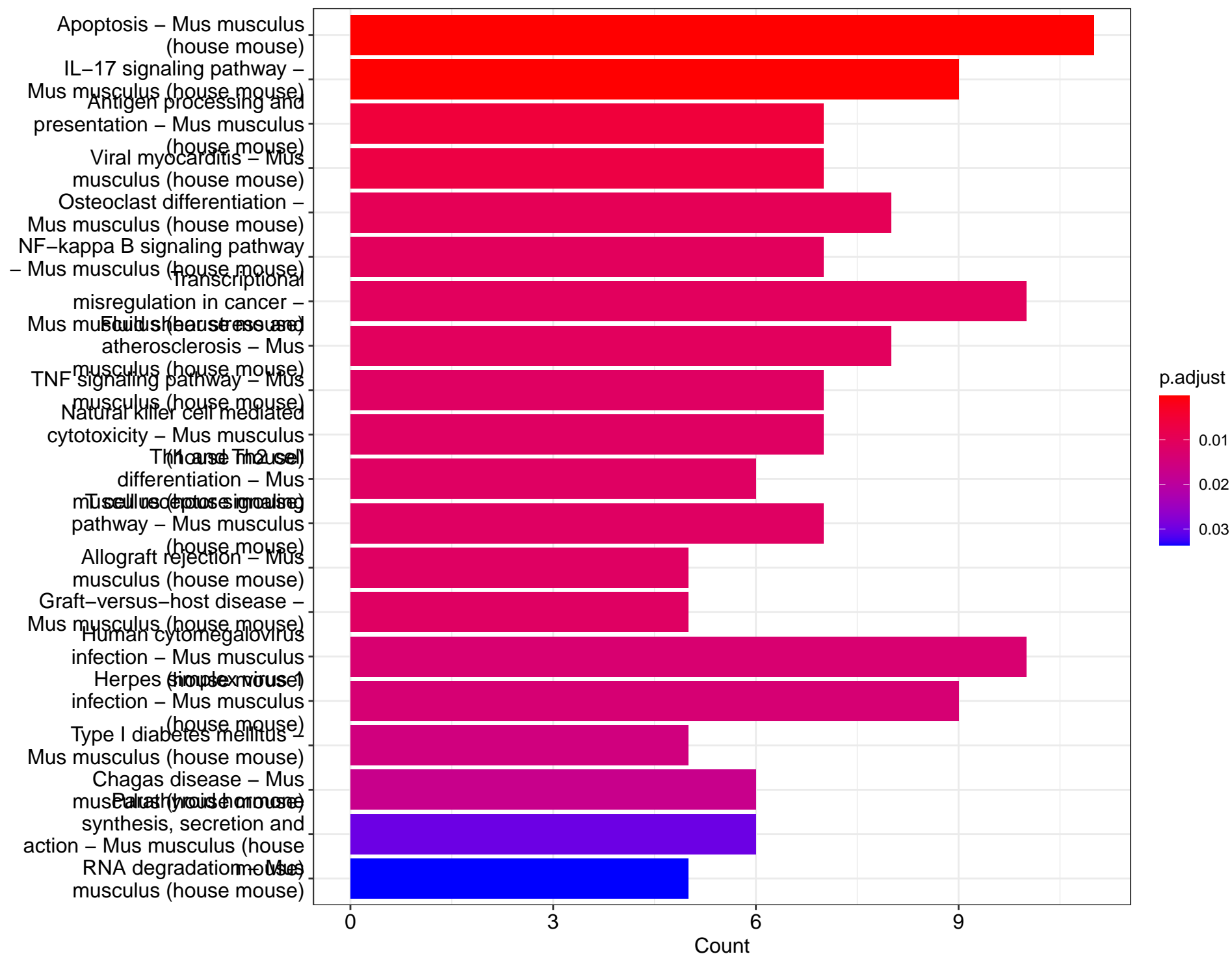

Supplement: Supplementary file 2 [file DataSheet2.zip › Supplementary Date 2/Enrichment_GO_KEGG_with_geneSymbol/CD8_Cytotoxic Ctrl vs 3mpi/3D-PBS vs 3M-A KEGG_visualization.pdf]

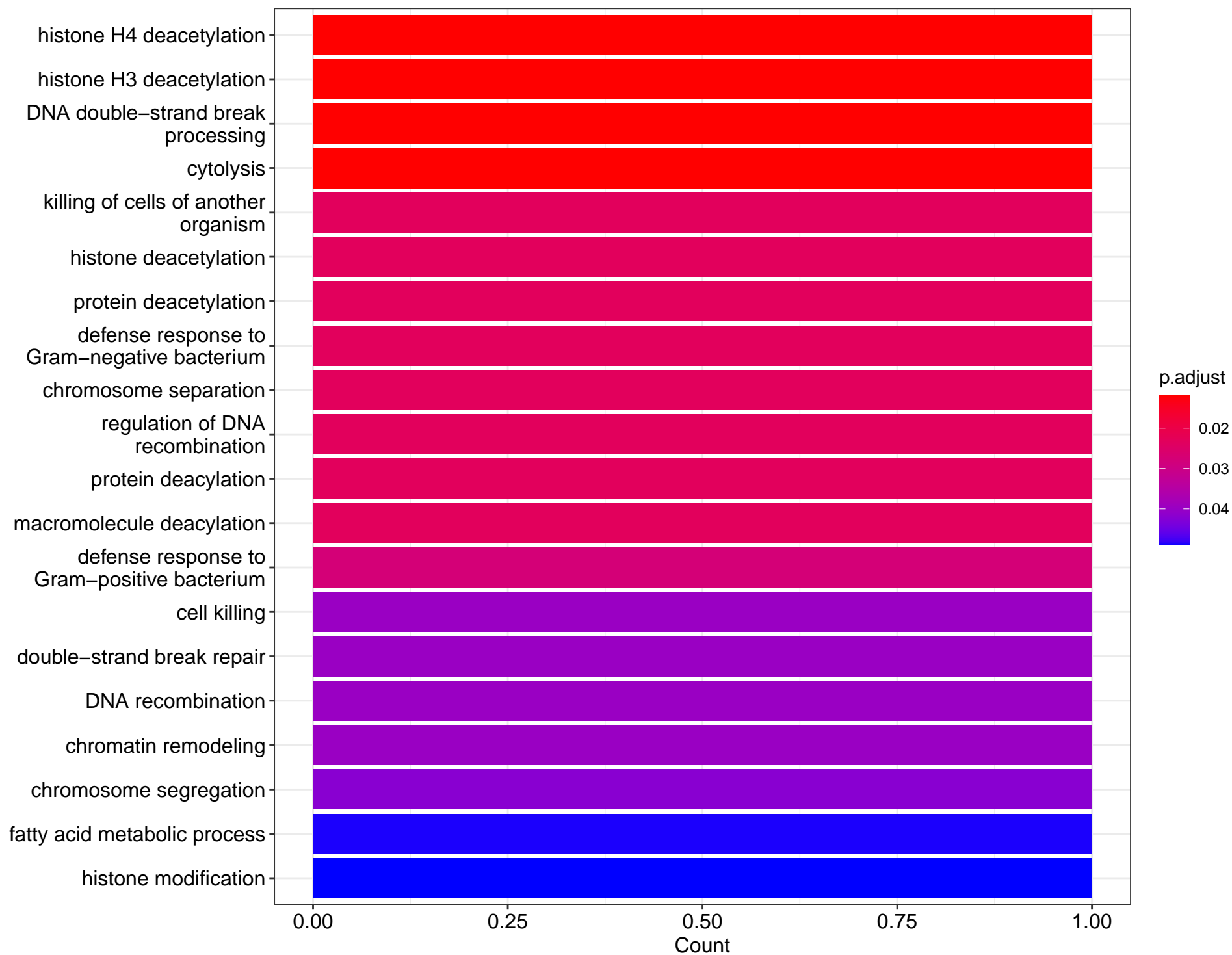

Supplement: Supplementary file 2 [file DataSheet2.zip › Supplementary Date 2/Enrichment_GO_KEGG_with_geneSymbol/CD8_Effector_Mmemory 3dpi vs 3mpi/GO_visualization.pdf]

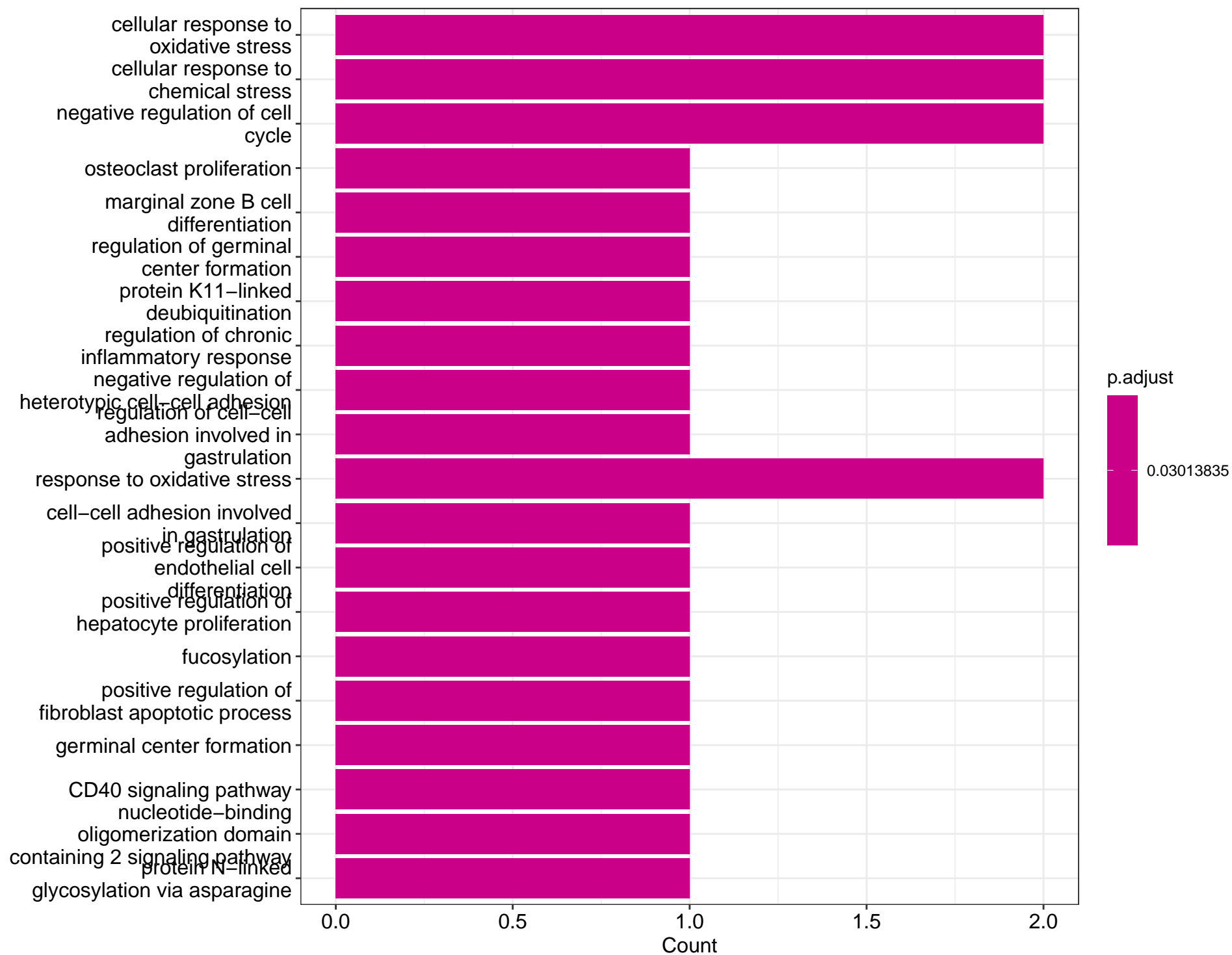

Supplement: Supplementary file 2 [file DataSheet2.zip › Supplementary Date 2/Enrichment_GO_KEGG_with_geneSymbol/CD8_Effector_Mmemory Ctrl vs 3mpi/GO_visualization.pdf]

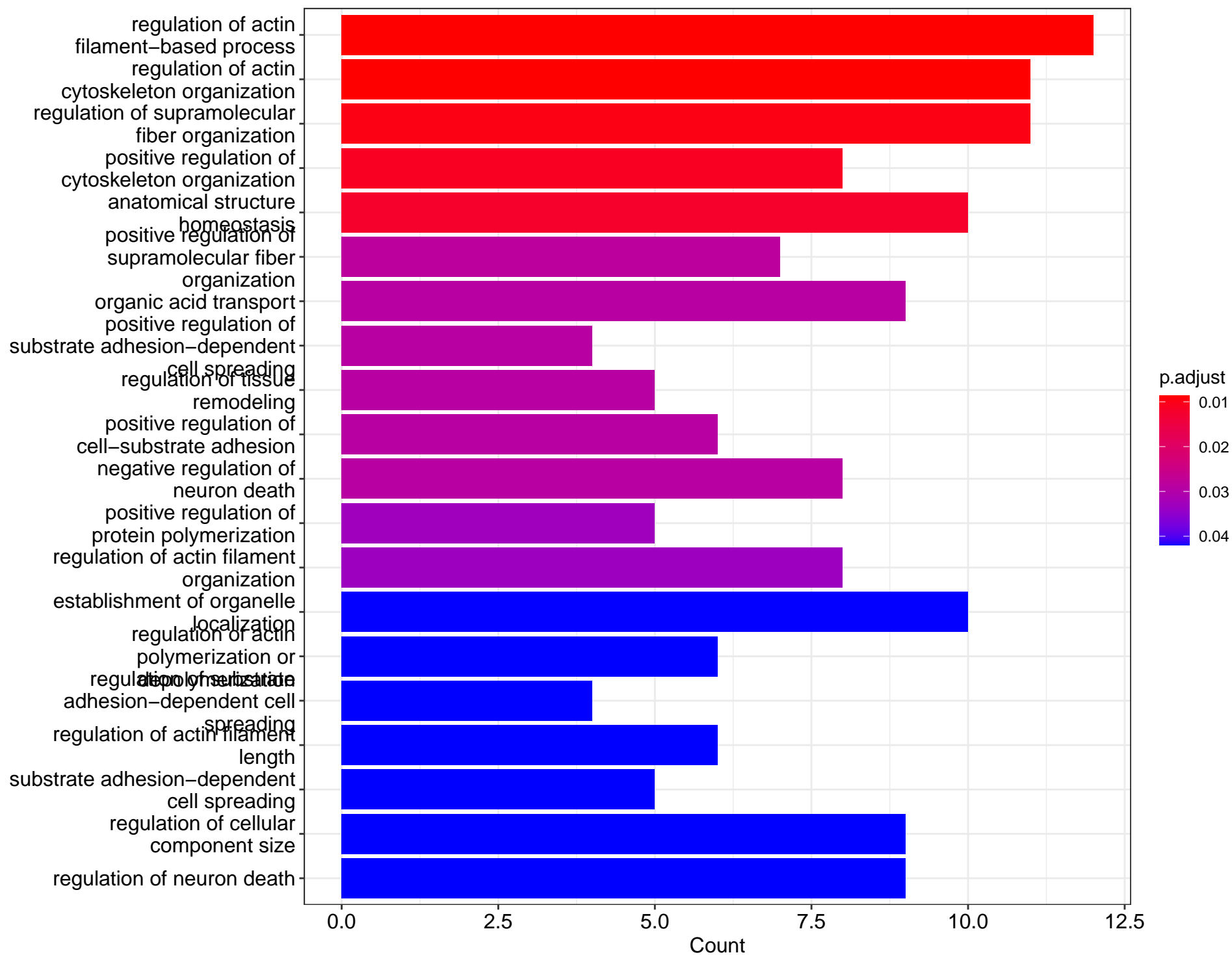

Supplement: Supplementary file 2 [file DataSheet2.zip › Supplementary Date 2/Enrichment_GO_KEGG_with_geneSymbol/CD8_Exhausted 3dpiA vs 3mpi/CD8_Exhausted 3D-A vs 3M-A GO_visualization.pdf]

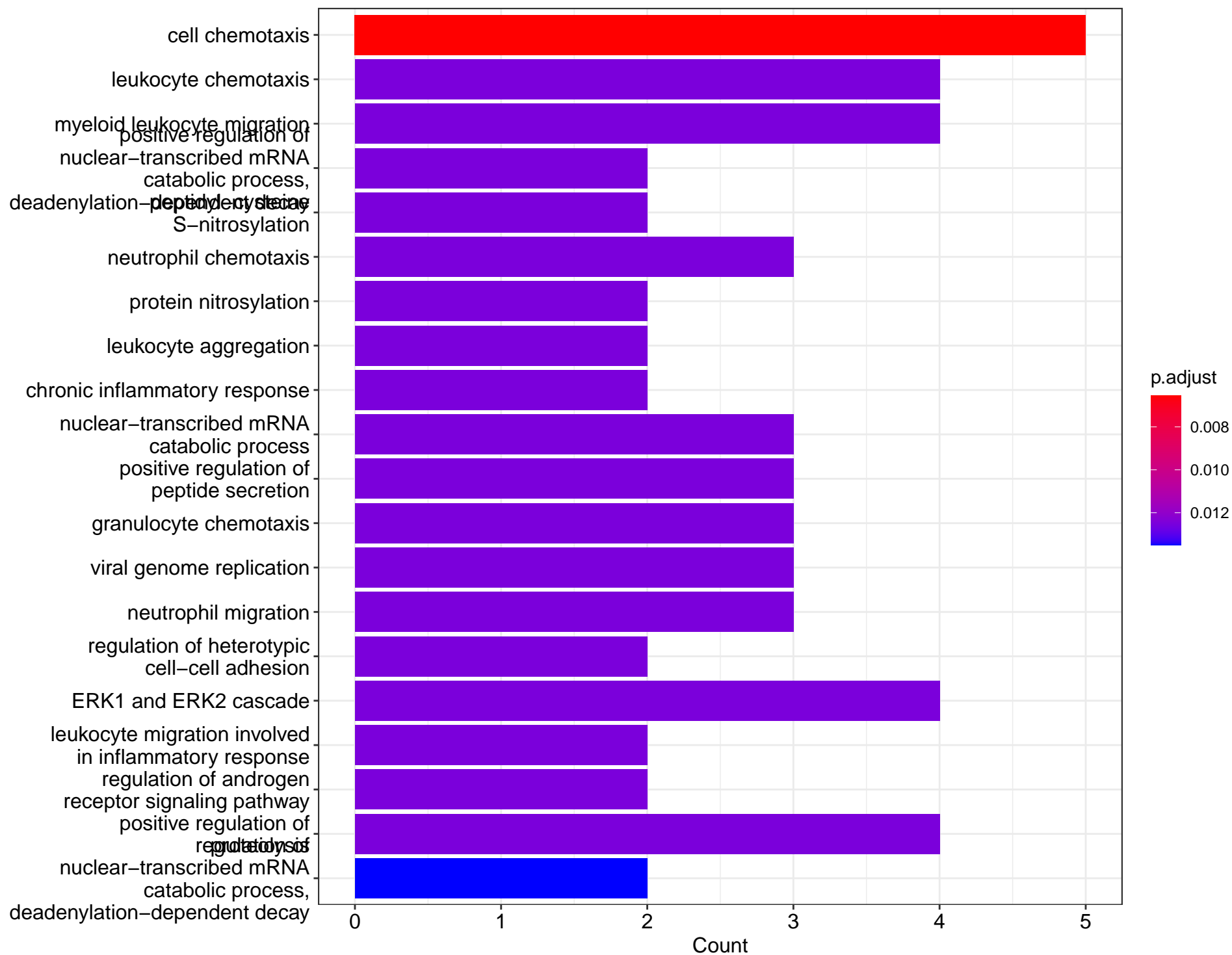

Supplement: Supplementary file 2 [file DataSheet2.zip › Supplementary Date 2/Enrichment_GO_KEGG_with_geneSymbol/CD8_Exhausted Ctrl vs 3mpi/CD8_Exhausted 3D-D(PBS) vs 3M-A GO_visualization.pdf]

IL-17 signaling pathway –  
Mus musculus (house mouse)

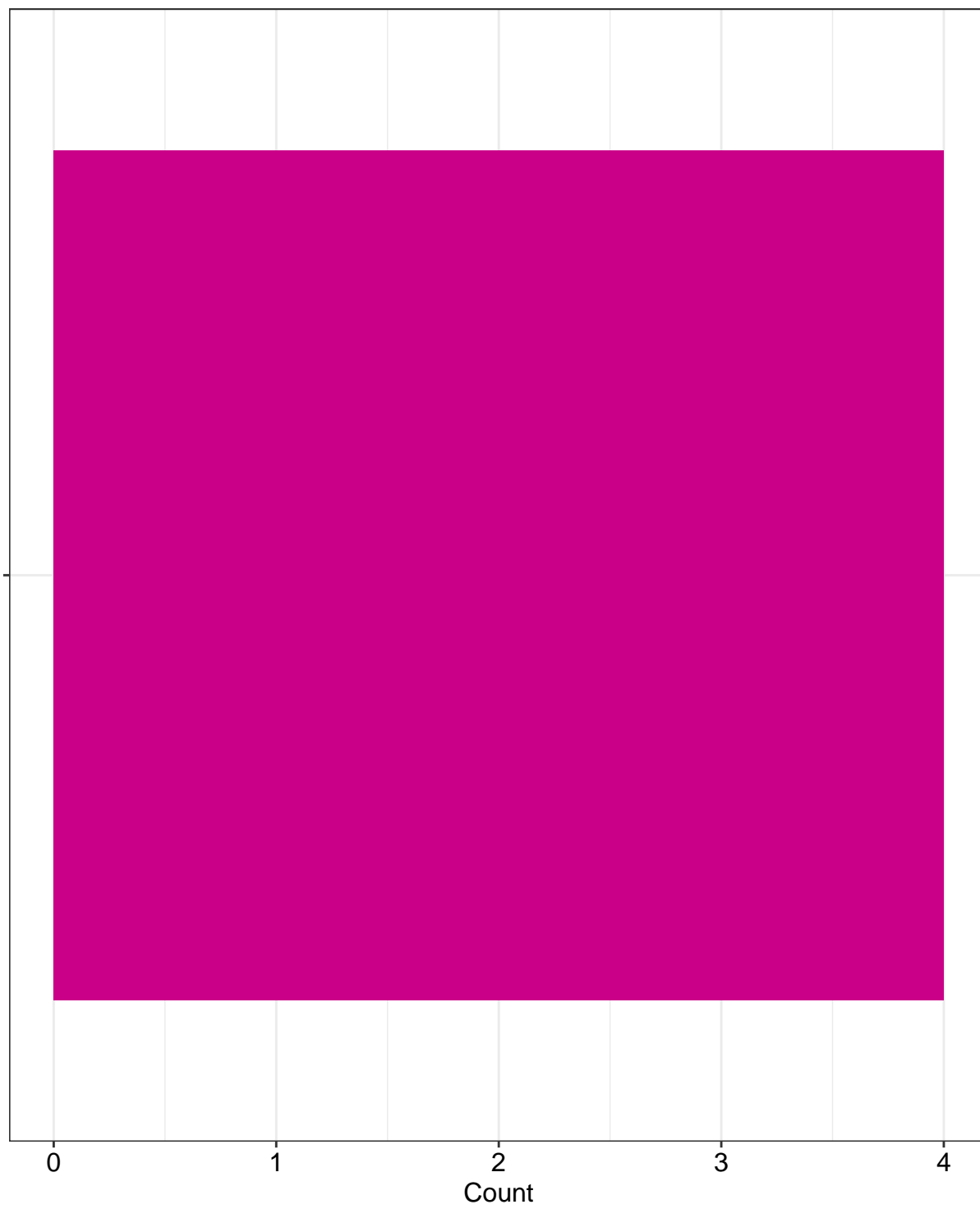

Supplement: Supplementary file 2 [file DataSheet2.zip › Supplementary Date 2/Enrichment_GO_KEGG_with_geneSymbol/CD8_Exhausted Ctrl vs 3mpi/CD8_Exhausted 3D-D(PBS) vs 3M-A KEGG_visualization.pdf]
