# Supplementary material for: Alveolar echinococcosis drives functional reprogramming of hepatic CD8+ T cells
Source: Front Cell Infect Microbiol. 2026 Feb 19;16:1747682. doi: 10.3389/fcimb.2026.1747682 (PMC12960575; doi:10.3389/fcimb.2026.1747682)
Supplement: Supplementary file 2 [file DataSheet2.zip › Supplementary Date 2/Enrichment_GO_KEGG_with_geneSymbol/CD8_Effector_Mmemory 3dpi vs 3mpi/KEGG_visualization.pdf]

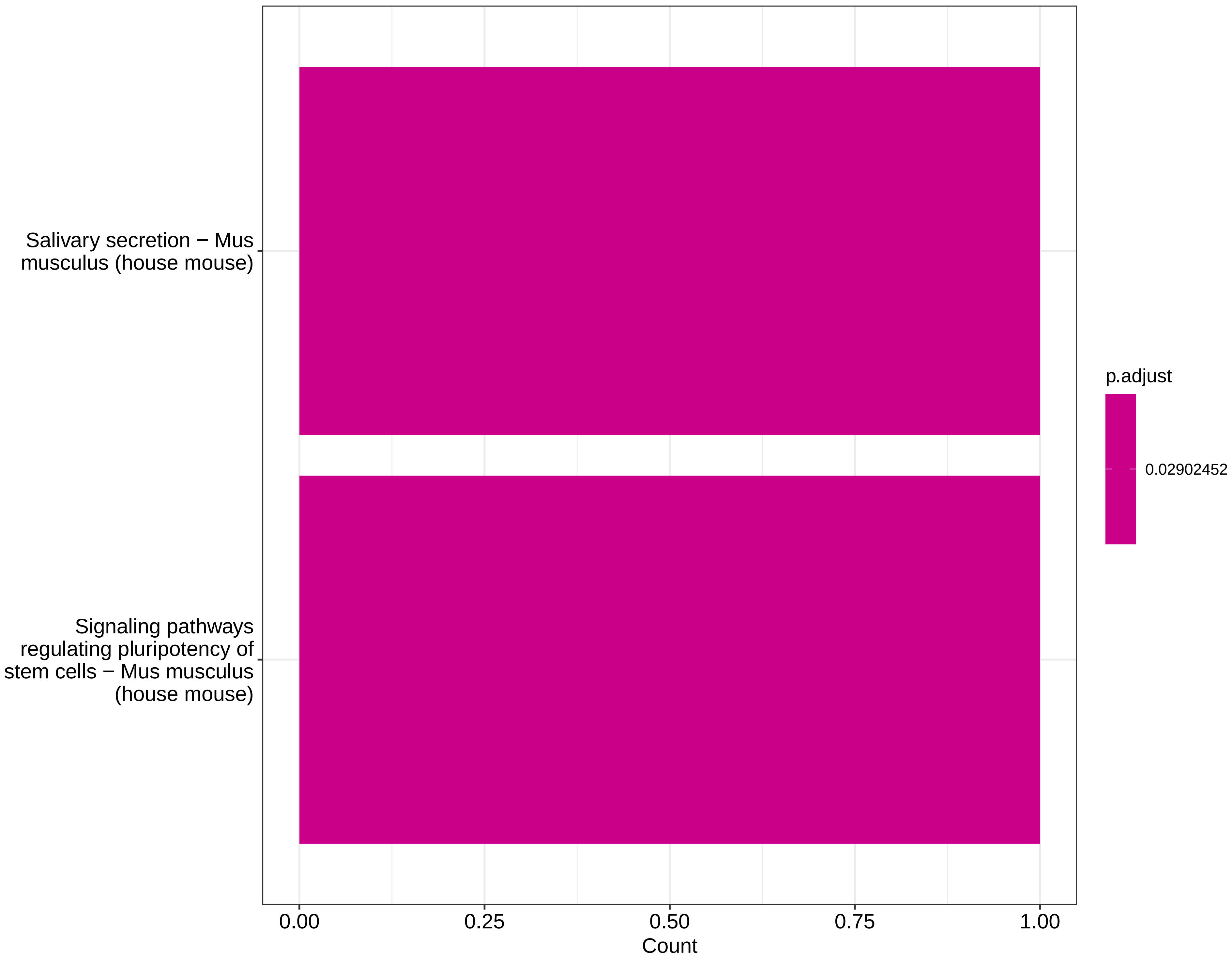
Salivary secretion − Mus musculus (house mouse)
Signaling pathways regulating pluripotency of stem cells − Mus musculus (house mouse)
0.00
0.25
0.50
0.75
1.00
Count
p.adjust
0.02902452
