# Supplementary material for: Alveolar echinococcosis drives functional reprogramming of hepatic CD8+ T cells
Source: Front Cell Infect Microbiol. 2026 Feb 19;16:1747682. doi: 10.3389/fcimb.2026.1747682 (PMC12960575; doi:10.3389/fcimb.2026.1747682)
Supplement: Supplementary file 2 [file DataSheet2.zip › Supplementary Date 2/Enrichment_GO_KEGG_with_geneSymbol/CD8_Effector_Mmemory Ctrl vs 3mpi/KEGG_visualization.pdf]

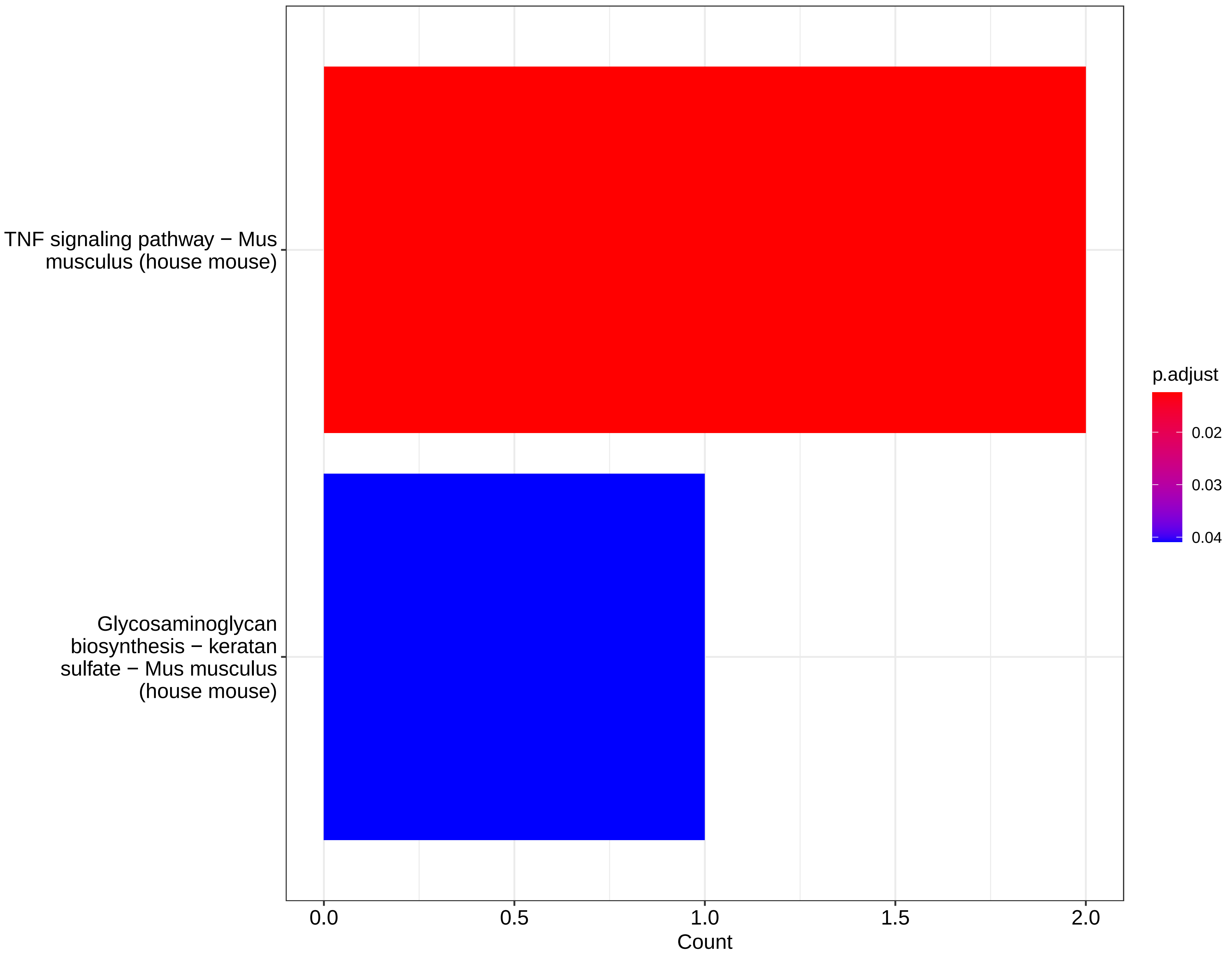

TNF signaling pathway − Mus musculus (house mouse)
Glycosaminoglycan biosynthesis − keratan sulfate − Mus musculus (house mouse)
0.0
0.5
1.0
1.5
2.0
Count
p.adjust
0.02
0.03
0.04
