# Supplementary material for: Alveolar echinococcosis drives functional reprogramming of hepatic CD8+ T cells
Source: Front Cell Infect Microbiol. 2026 Feb 19;16:1747682. doi: 10.3389/fcimb.2026.1747682 (PMC12960575; doi:10.3389/fcimb.2026.1747682)
Supplement: Supplementary file 3 [file DataSheet3.pdf]

Supplementary Material

1 **Supplementary Table S1 . HE Scoring Criteria**

Category	Score	Description
Inflammatory infiltration	0	No or minimal inflammatory cells, <100/HPF
	1	Mild, focal or sparse distribution, ~100–500/HPF
	2	Moderate, band-like accumulation, ~500–1000/HPF
	3	Severe, diffuse dense infiltration, >1000/HPF
Sinusoidal dilation & congestion	0	No dilation or congestion
	1	Mild, <25% of field
	2	Moderate, 25–50% of field, widened sinusoids
	3	Severe, >50% of field, marked erythrocyte accumulation
Hepatocellular necrosis	0	No identifiable necrotic area
	1	Mild focal/spotty necrosis, <5% of field
	2	Moderate patchy necrosis, ~5–30% of field
	3	Severe, >30% of field, large parenchymal loss
Fibrosis	0	Only delicate fine fibers
	1	Mild, focal or scattered thin fibrous strands
	2	Moderate, extended fibrous septa
	3	Severe, thick bridging fibrosis or pseudo-lobule formation

2 Supplementary Table S2. Specific information on flow cytometry antibodies

Marker	Fluorochrome	Clone	Catalog No.	Vendor	Dilution
Fixable Viability Stain 510	BV510	N/A	564406	BD Horizon	1:1000
CD45	BV786	I3/2.3	752409	BD OptiBuild	1:100
CD3e	PE-CF594	145-2C11	562286	BD Horizon	1:100
CD4	BV480	H129.19	746631	BD OptiBuild	1:100
CD8	Pacific Blue	53-6.7	558106	BD Pharmingen	1:100
Ly-6G	FITC	1A8	551460	BD Pharmingen	1:100
I-A/I-E	PE	M5/114.15.2	562010	BD Pharmingen	1:100
F4/80	BV421	T45-2342	565411	BD Horizon	1:100
CD11b	APC	M1/70	553312	BD Pharmingen	1:100
CD11c	BV650	HL3	564079	BD Horizon™	1:100
CD62L	PerCP-Cy5.5	MEL-14	560513	BD Horizon	1:100
CD44	BUV496	IM7	569706	BD Horizon	1:100
PD-1	APC-R700	J43	565815	BD Horizon	1:100
Granzyme B	eFluor™ 660	NGZB	50-8898-80	eBioscience	1:100
anti-CD28	N/A	37.51	102115	BioLegend	1:100

3 **Supplementary Table S3. Flow cytometry gating strategy and phenotype definitions**

Population / readout	Parent gate	Positive markers (+)	Negative markers (−)	Notes
Leukocytes	Singlets, live cells	CD45+	—	Viability dye used to exclude dead cells
T cells	CD45+	CD3e+	—	Surface staining
CD4 T cells	CD45+ CD3e+	CD4+	CD8−	Surface staining
CD8 T cells	CD45+ CD3e+	CD8+	CD4−	Surface staining
CD8 Tem	CD45+ CD3e+ CD8+	CD44$^{\wedge high}$CD62L$^{\wedge low}$	—	Memory phenotyping (surface)
Exhaustion-like CD8 (PD-1+)	CD45+ CD3e+ CD8+	PD-1+	—	Surface staining，PD-1 was used as an exhaustion-associated marker; analyzed within CD8 gate.
Cytotoxic CD8 (GZMB+)	CD45+ CD3e+ CD8+	GZMB+	—	Intracellular; after ex vivo stimulation and secretion blocking
Macrophages	CD45+	F4/80+ CD11b+	Ly6G−	Surface staining; neutrophils excluded by Ly6G−
Dendritic cells (DCs)	CD45+	CD11c+ I-A/I-E+	Ly6G−	Surface staining; granulocytes excluded by Ly6G−

4 Supplementary Figures S1

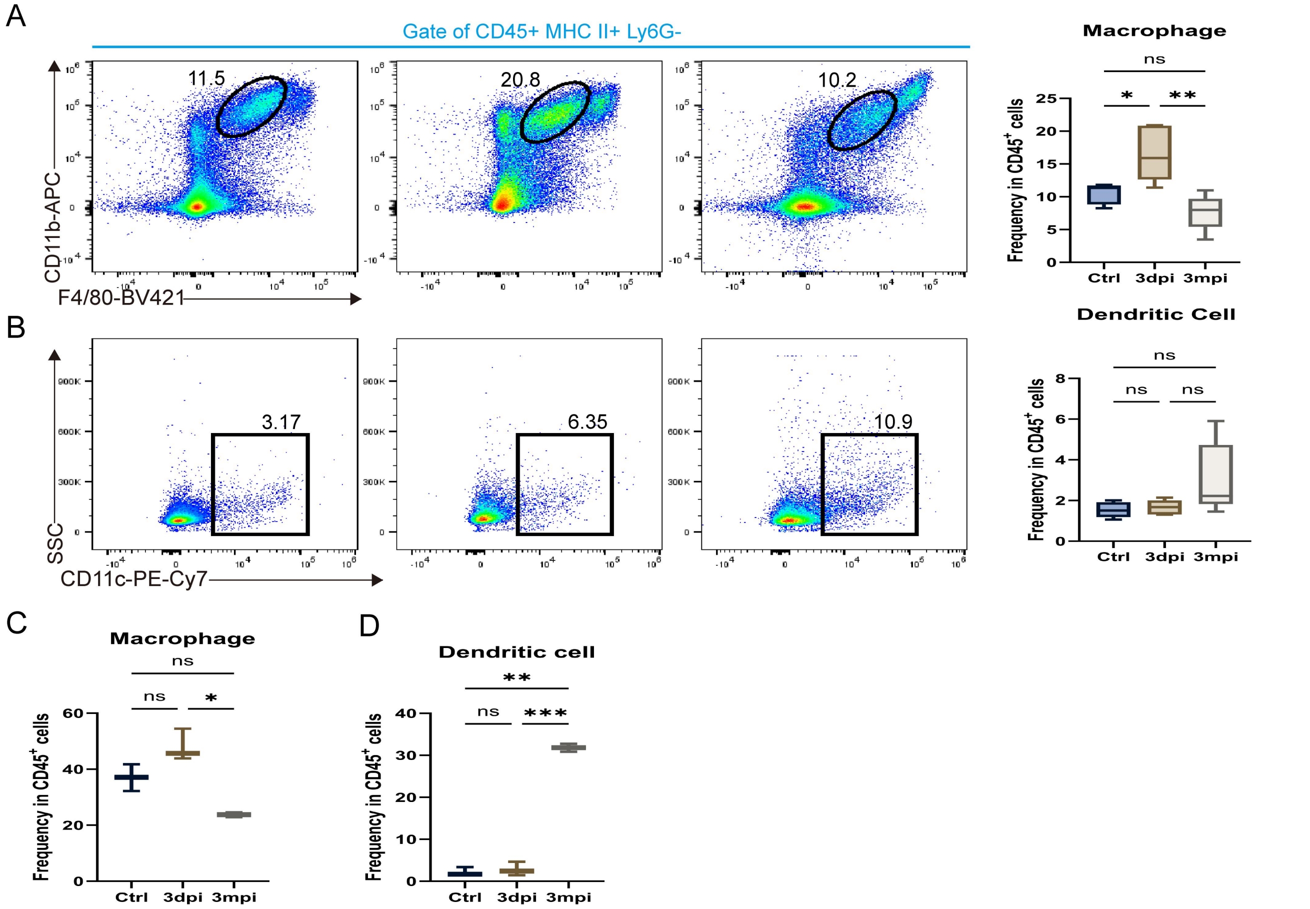

Supplementary Figure S1. Flow cytometric validation and single-cell quantification of macrophage and dendritic cell frequencies. (A) Representative flow-cytometry plots showing macrophage gating ($CD11b^+F4/80^+$) within $CD45^+MHC\ II^+Ly6G^-$ liver immune cells in control, 3 dpi, and 3 mpi groups, with corresponding frequencies shown at right. (B) Representative flow-cytometry plots showing dendritic cell gating ($CD11c^+$) within $CD45^+MHC\ II^+Ly6G^-$ liver immune cells in control, 3 dpi, and 3 mpi groups, with corresponding frequencies shown at right. (C) Proportion of macrophages derived from single-cell RNA-seq dataset across control, 3 dpi, and 3 mpi groups. (D) Proportion of dendritic cells derived from single-cell RNA-seq dataset across control, 3 dpi, and 3 mpi groups.

5 Supplementary Figures S2

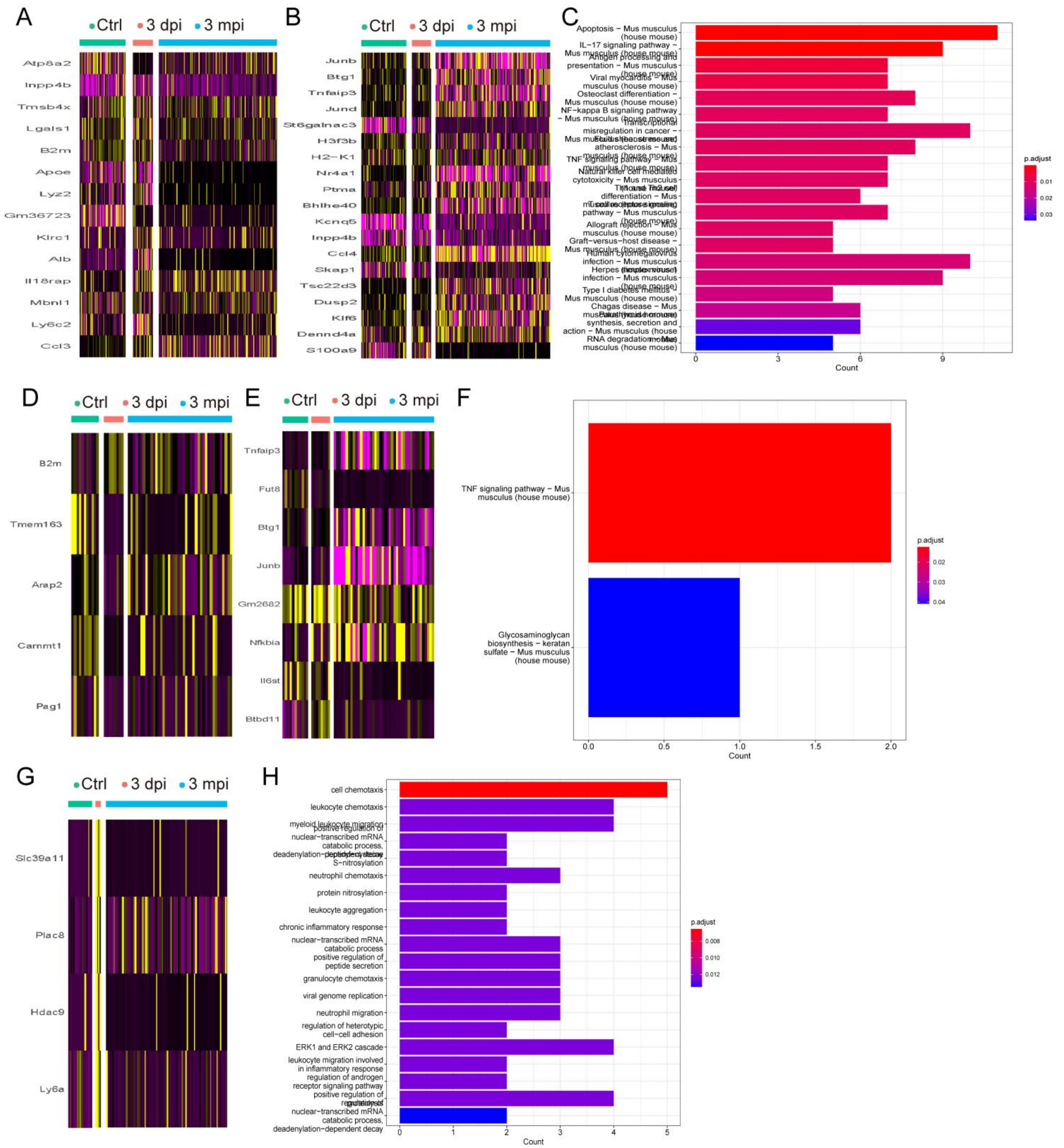

Supplementary Figure S2. Differential expression and pathway enrichment analyses of CTLs and Tem subsets. (A) Heatmap showing differentially expressed genes (DEGs) of CTLs from the Ctrl vs 3 dpi comparison. (B) Heatmap showing DEGs of CTLs from the Ctrl vs 3 mpi comparison. (C) KEGG pathway enrichment bubble plot based on CTL DEGs from the Ctrl vs 3 mpi comparison. (D) Heatmap showing selected differentially expressed genes (DEGs) of Tem cells from the Ctrl vs 3 dpi comparison. (E) Heatmap showing selected DEGs of Tem cells from the Ctrl vs 3 mpi comparison. (F) KEGG pathway enrichment bubble plot based on Tem DEGs from the Ctrl vs 3 mpi comparison.

(G) Heatmap showing differentially expressed genes (DEGs) of Tem cells from the Ctrl vs 3 dpi comparison. (H) GO pathway enrichment bubble plot based on Tem DEGs from the Ctrl vs 3 mpi comparison.
